# Probabilistic Admission Control with Dual-Window Channel Occupancy Statistics for Statistical Priority-Based Multiple Access

**DOI:** 10.3390/s26196347

**Published:** 2026-10-08

**Authors:** Yu Wu, Byung-Seo Kim

**Affiliations:** 1 School of Computer Science, Shandong Xiehe University, Jinan 250109, China; wuyu1@sdxiehe.edu.cn; 2 Department of Software & Communications Engineering, Hongik University, Sejong City 30016, Republic of Korea

**Keywords:** statistical priority-based multiple access, priority threshold, channel occupancy statistics, probabilistic admission control, dual-window

## Abstract

Statistical priority-based multiple access (SPMA) provides differentiated channel access for multi-priority traffic by comparing the channel occupancy statistic (COS) with hierarchical thresholds. However, existing threshold-setting methods ignore the inherent fluctuation in the COS caused by random traffic arrivals, so that a large fraction of time slots are incorrectly blocked and the channel performance degrades sharply under overload. To address this issue, this paper proposes DW-PA-SPMA, a dual-window probabilistic admission SPMA protocol. Its core idea is to move traffic management from post-hoc threshold interception at the transmission time slot to probabilistic admission at the packet generation instant: based on a capacity budget that relates the available channel capacity to the traffic demand of each priority, excess packets are truncated at the source following the principle of “highest priority first, remaining capacity for lower priorities”, so that no queue buffers or channel contention resources are wasted. A dual-window statistic separates instantaneous fluctuation from the steady-state trend, and a dual-EWMA difference method estimates the load variation time scale online, allowing the long window length to adapt to traffic variations without prior load information. Simulation results under a six-segment time-varying load show that the protocol keeps the highest-priority end-to-end completion rate above the 90% target from light load to full load and remains stable under 2.0× overload, whereas existing threshold-based protocols degrade sharply in the heavy-load segment, demonstrating the advantages of the proposed protocol in dynamic traffic scenarios.

## 1. Introduction

In high-speed dynamic wireless scenarios such as swarm unmanned aerial vehicle (UAV) ad hoc networks and vehicular mobile Ad Hoc networks, multiple heterogeneous services coexist, including delay-sensitive control messages, environmental sensing data packets, and large-capacity multimedia transmissions. Different services have differentiated quality of service (QoS) requirements for transmission delay, packet reliable reception probability, and channel resource occupancy [1,2,3]. To this end, various multiple access schemes have been proposed, whose mechanism features and applicable conditions differ from each other. Time division multiple access (TDMA) allocates non-overlapping time slots to each node to achieve collision-free transmission, but relies on static time-slot pre-allocation and is difficult to adapt to bursty data flows [4]. Carrier sense multiple access (CSMA) only distinguishes between the idle and busy binary states of the channel, and under high load the packet collision probability rises sharply and the reliable transmission performance degrades precipitously [5]. Non-orthogonal multiple access (NOMA) and orthogonal frequency division multiple access (OFDMA) realize multi-user access through resource partitioning in the power domain and the frequency domain, respectively [6,7]; their typical deployment relies on a base station or a central control node for resource allocation and scheduling, and it is difficult for them to adapt to centerless distributed network topologies. Therefore, none of the above mechanisms can effectively cope with the differentiated requirements of multiple priority services in centerless, highly dynamic networks. Statistical Priority-based Multiple Access (SPMA), as a contention-based medium access control (MAC) protocol for distributed networks, relies on the channel occupancy statistic (COS) and hierarchical access thresholds to achieve distributed channel contention management without a central scheduling node. It can guarantee the transmission reliability of high-priority packets while taking into account the overall channel utilization, and is currently the mainstream access technology for heterogeneous data link networks, cluster UAV networking and other scenarios [8,9,10].

**Figure 1:**
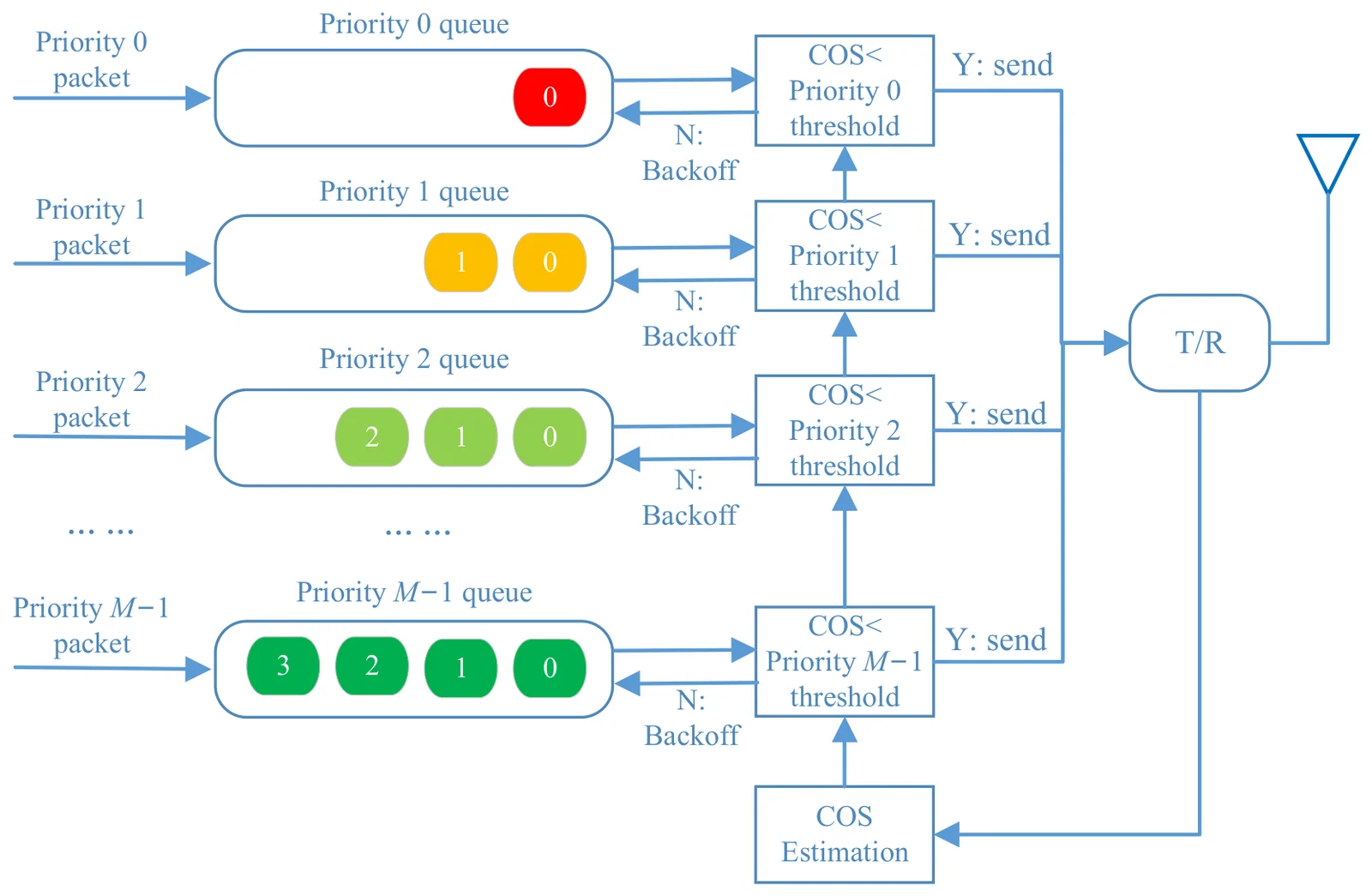
Overall framework of the existing SPMA protocol.

The overall framework of the SPMA protocol is shown in Figure 1. Its core scheduling mechanism is as follows: nodes continuously count the number of data packets sent to the channel within the detection window to obtain the real-time COS value, and a packet can be transmitted only when the current COS is less than the threshold corresponding to its priority; otherwise, graded random backoff is executed. The priority threshold is the core control parameter of SPMA channel access, directly determining the channel access rights of each service and profoundly affecting the packet transmission success rate, queuing delay, and overall network throughput. A reasonable threshold management mechanism must simultaneously achieve two objectives: first, stably maintaining the preset reliable transmission indicators for high-priority services under all time-varying traffic scenarios including light load, full load, and continuously varying overload; second, providing sufficient access opportunities for low-priority services when channel idle capacity exists, avoiding long-term channel idleness and resource waste. Existing research on priority thresholds has mainly focused on optimal threshold value selection and dynamic adjustment methods.

However, this study reveals that existing threshold-setting methods suffer from systematic deficiencies that severely suppress the transmission of normal highest-priority traffic and fail to effectively utilize idle channel time to improve the service quality of other priority services. Specifically, existing methods generally set the highest-priority threshold to the mean COS, but neglect the inherent volatility of COS around its mean caused by the randomness of traffic arrivals. Even when the highest-priority traffic is theoretically at full load, nearly half of the time slots have instantaneous COS exceeding the mean, causing packets to be incorrectly blocked and the transmission success rate to fall far below the preset target. This paper further discovers that the channel access control mechanism based solely on threshold constraints and random backoff at the time-slot decision stage has inherent defects and cannot effectively achieve controlled and efficient channel resource utilization. To this end, this paper steps outside the existing SPMA research framework and proposes a dual-window probabilistic admission SPMA protocol (DW-PA-SPMA), which enables multiple priority services to obtain better QoS under various scenarios including light load, full load, and overload.

The main contributions of this paper include the following three aspects:

1. Revealing the systematic failure mechanism of the existing SPMA threshold mechanism: pointing out that after setting the highest-priority threshold to the mean COS, the inherent fluctuation in COS causes nearly half of the time slots to incorrectly block packets, and the post-hoc threshold interception mode inevitably leads to queue accumulation and aggregated collisions, which is a deeper structural problem than threshold value deviation;
2. Establishing a source-end probabilistic admission control framework based on capacity budget: shifting traffic control from the post-hoc decision at the transmission time slot to the packet generation source, deriving the analytical relationship among available channel capacity, priority service demand, and admission probability, enabling the channel load under overload scenarios to be theoretically calculable and controllable;
3. Designing a complete protocol implementation with dual-window statistics and adaptive long-window adjustment: the short window captures instantaneous occupancy while the long window extracts steady-state load to separate fluctuation from trend, and introducing the dual-EWMA difference method for online estimation of the load variation time scale, enabling the long window length to dynamically adjust with traffic variations without requiring prior load information.

The remainder of this paper is organized as follows: Section 2 reviews related work; Section 3 presents the system model and research motivation; Section 4 describes in detail the modular architecture, core mechanisms, and implementation flow of the DW-PA-SPMA algorithm; Section 5 verifies the algorithm performance through simulation experiments; Section 6 discusses algorithm advantages, applicability, and future work; and Section 7 concludes the paper.

## 2. Related Work

### 2.1. Baseline Threshold Determination Methods

The basic approach of existing SPMA priority threshold design schemes is to first select the highest priority (or in a few cases, the lowest priority) as the reference priority, determine a baseline threshold, and then determine thresholds for other priorities according to certain principles. The baseline threshold can be determined using analytical methods or measurement-based methods.

A typical analytical method was proposed in [11]. This method assumes that packets of different services are randomly sent to multiple channels according to independent Poisson distributions, and derives the transmission success rate formula on a single channel:

(1) $$P_{s}=\sum_{i=N_{b}/2}^{N_{b}}C_{N_{b}}^{i}\cdot {{\left(P_{\mathrm{bs}}\right)}^{i}\cdot (1-P_{\mathrm{bs}})}^{N_{b}-i},$$

where *N_b_* is the number of pulses into which a data packet is split during physical layer transmission, and *P_bs_* is the pulse transmission success rate. The physical meaning of Formula (1) is that as long as the receiving end successfully receives no fewer than half of the pulses, the entire packet can be correctly reassembled. Setting the target transmission success rate *P_succ_* for the reference priority, the corresponding pulse transmission success rate $P_{\mathrm{bs}}^{*}$ when *P_s_* = *P_succ_* can be obtained from Formula (1), and then the baseline channel occupancy rate *COS_base_* that satisfies the reference priority target transmission success rate, i.e., the reference priority threshold, can be obtained according to:

(2) $${\mathrm{COS}}_{\mathrm{base}}=\frac{-W\cdot N_{f}\cdot \ln P_{\mathrm{bs}}^{*}}{N_{b}},$$

where *W* is the detection window length, and *N_f_* is the number of available channels. Formula (2) reveals the logarithmic relationship between the baseline channel occupancy rate and the target transmission success rate: the higher the target transmission success rate *P_succ_*, the closer $P_{\mathrm{bs}}^{*}$ is to 1, and the smaller *COS_base_* becomes.

A typical measurement-based method is described in [9,12,13]: configure a fully connected network with only the reference priority service, where the offered traffic load *TR* of this service gradually increases from a small value, while the ALOHA protocol is used at the MAC layer so that packets are transmitted upon arrival. During this process, at any node, the detection window is used to count in real time the packet transmission success rate (number of packets correctly received by the receiver divided by the total number of packets generated by the service) and the number of packets on the channel. When the transmission success rate decreases from 100% to the preset target transmission success rate *P_succ_*, the mean of the packet counts from all detection windows is calculated, and this value is taken as the reference priority threshold, i.e., *COS_base_*. The current traffic load is the reference traffic load that guarantees the target transmission success rate, denoted as *TR_base_*.

It should be noted that the ALOHA scenario is introduced in this paper only for calibrating the baseline threshold and for explaining the statistical relationship between packet arrivals and channel occupancy on a shared channel, serving as a baseline for the subsequent analysis of the SPMA mechanism. The protocol proposed in this paper and all performance comparisons are based on the SPMA framework and do not adopt the ALOHA protocol; ALOHA serves here as an analysis and calibration tool rather than an actually deployed protocol.

After determining the initial thresholds for each priority according to the above schemes, based on whether the thresholds can be adaptively updated in real time with network load and topology changes, they can be further divided into two technical routes: static threshold schemes and dynamic threshold schemes. Each route contains multiple representative improved algorithms, whose core design ideas, advantages, and inherent limitations are reviewed separately below.

### 2.2. Static Threshold Setting Methods

The core feature of static threshold schemes is that all priority thresholds are solved offline once during the network initialization phase, and the threshold values remain fixed throughout network operation, unable to adjust with traffic load or network environment changes.

In the static threshold category, the schemes can be grouped by their technical routes. One route relies on queueing-theoretic analysis: the scheme in [12] solves the highest-priority baseline threshold offline under the constraint of a preset transmission success rate using the M/G/1 queueing model and the multi-channel pulse interference collision model, and decreases the low-priority thresholds layer by layer with fixed constant differences; the scheme in [14] establishes an SPMA mathematical analysis model based on M/G/1 priority queues and derives analytical expressions for the successful reception probabilities of single pulses and data packets, also using fixed differences to configure the low-priority thresholds. Schemes of this route have complete theoretical derivation support but are only suitable for steady-state scenarios with long-term stable traffic. Another route improves the observation accuracy of the channel load: the scheme in [15] introduces a bidirectional long short-term memory network (BiLSTM) to correct physical layer pulse statistical errors and uses historical COS time series to predict the current channel load, but the thresholds at all levels are still statically configured offline, so only the load observation accuracy is optimized without solving the threshold adaptation problem, representing a typical “time series prediction + fixed threshold” improvement route. A further route represents the general engineering implementation approach for early SPMA static thresholds: the scheme in [16] proposes a dedicated SPMA networking scheme for FANETs, first calibrating the lowest-priority threshold through single-hop simulation and then deriving the higher-level thresholds according to traffic proportion. In addition, the scheme in [17] derives an analytical expression for the single-pulse successful transmission probability in multi-channel half-duplex systems, gives an analytical expression for the highest-priority threshold, and configures the remaining thresholds with equal arithmetic decrements, also without introducing a dynamic adjustment mechanism; the scheme in [13] proposes a dual-bound threshold setting method based on ALOHA scenario testing, which sets the load corresponding to a 95% transmission success rate as the highest threshold and the load corresponding to 99% as the lowest threshold, with intermediate thresholds linearly interpolated, and proposes probabilistic access control for low-priority data, but the thresholds themselves remain fixed after initialization.

The common limitation of the above methods is that once the thresholds are determined, they no longer adjust with network load changes. In continuously time-varying traffic scenarios, either the high-priority success rate falls below the target or channel resources remain idle for extended periods.

### 2.3. Dynamic Threshold Adjustment Methods

Dynamic threshold schemes can update the thresholds at all priority levels in real time according to changes in network topology, node rates, and traffic load. Based on differences in the triggering adjustment mechanism, they can be further divided into two subcategories: event-triggered dynamic thresholds and load-feedback closed-loop dynamic thresholds.

The event-triggered dynamic threshold schemes recalculate the thresholds only when network parameters undergo a jump. The scheme in [18] proposes an event-driven adaptive threshold strategy, deriving the analytical relationship between transmission success rate and traffic load based on the M/G/1 queueing model and the pulse collision model, and recalculating all thresholds when the node transmission rate ratio changes; the scheme in [19] belongs to the same category, recalculating thresholds only when the node rate ratio or the network topology undergoes a jump. However, schemes of this type update the thresholds only when topology parameters jump, and lack the ability to regulate continuously varying loads. The load-feedback closed-loop dynamic threshold schemes attempt to overcome this limitation from different angles. To address the hidden terminal problem in multi-hop environments, the scheme in [20] proposes a cross-layer optimized distributed channel load sensing algorithm that uses the ratio of single-hop to two-hop neighbors to dynamically correct fixed thresholds, but the correction factor depends only on topological density parameters and does not introduce direct performance feedback such as channel load or transmission success rate. The scheme in [21] transforms discrete priorities into a continuous characterization system based on delay QoS exponents, calculating the minimum supportable delay QoS exponent in real time according to the channel occupancy, so that the thresholds can vary dynamically with the load, but it focuses on innovating the priority characterization method. The scheme in [22] proposes a load-feedback closed-loop dynamic threshold scheme, iteratively correcting thresholds based on the overall transmission success rate of mixed packets across the entire network, but it cannot isolate the independent load of the highest priority and suffers from convergence oscillation and adjustment lag. In addition, the scheme in [23] indirectly achieves dynamic regulation from the perspective of backoff mechanism optimization, constructing a backoff time model using a logarithmic growth function, but does not directly adjust the threshold values.

### 2.4. Network Performance Optimization Research for UAV Ad Hoc Networks

In recent years, research on network performance optimization in UAV ad hoc networks has mainly focused on three directions. The first is clustering and resource scheduling with age of information (AoI) as the optimization objective, which reduces information staleness through clustering structures, cluster-head selection, and transmission scheduling [24,25]. The second is the joint design of transmission and trajectory in UAV-assisted wireless powered communication networks (WPCNs), where the transmission power, time-slot allocation, and flight trajectory are jointly optimized under onboard energy constraints [26]. The third consists of other recent works on the performance modeling and optimization of UAV ad hoc networks, including related studies published in journals such as *IEEE TVT*. Most of these works seek performance improvement at the network layer or the system layer (clustering, trajectory, power, energy), and their premise is usually that the access behavior at the MAC layer is predictable. In contrast, the work of this paper is located at the MAC layer and addresses the underlying problem that access behavior becomes unpredictable under overload. When the reliability of the access layer cannot be guaranteed, the actual gains obtainable from upper-layer trajectory optimization, clustering, and AoI optimization are severely limited by packet loss and collisions at the access layer. Therefore, the MAC-layer admission control improvement of this paper and the above network-layer optimization works are complementary at the technical level, and their combination can form a more complete performance guarantee scheme for UAV ad hoc networks.

### 2.5. Methods Based on Artificial Intelligence and Machine Learning

Artificial intelligence (AI) and machine learning (ML) methods have been widely used in the design and optimization of MAC protocols in recent years, and can be roughly divided into three categories according to their technical routes. The first is access decision methods based on reinforcement learning and deep reinforcement learning, which model the adjustment of time-slot selection, backoff window length, or access probability as a sequential decision problem and learn policies by interacting with the environment using throughput, delay, or collision rate as the reward function. The second is load or channel state prediction methods based on time series forecasting, which adopt long short-term memory (LSTM), bidirectional LSTM, gated recurrent unit (GRU), or Transformer models to predict channel occupancy or traffic intensity and provide prediction inputs for threshold decisions or resource allocation. The third is service identification and scheduling methods based on classification, clustering, and learning, which identify service types from traffic features and accordingly assign access priorities or resources. Related studies [27,28,29,30] have achieved notable performance improvements in typical scenarios, and their common feature is reliance on data-driven model training and inference.

The method proposed in this paper differs from the above AI/ML methods in four respects. First, the optimization objective differs: the objectives of AI/ML methods are implicit in the reward or loss function and generally provide no analytical guarantee of the performance bound, whereas the admission probability in this paper is given directly by the capacity budget formula as an analytical solution without training, and the performance bound can be derived from the formula. Second, the online adaptation mechanism differs: AI/ML methods rely on online data to update model parameters and require data accumulation and training overhead, whereas this paper only estimates the time constant of a first-order statistic online, with no model parameters or offline dataset. Third, the computational and storage overhead differs: AI/ML methods need to store model parameters and perform matrix operations or recurrent neural network inference, whereas the proposed method only needs to maintain two window counters and two EWMA smoothing quantities for each priority, with a constant-level per-slot computational cost. Fourth, the access decision mechanism differs: the decision process of AI/ML methods is opaque, whereas the decision in this paper is given by an explicit deterministic formula chain in which every step can be independently verified and debugged. The two are not mutually exclusive: AI/ML methods can be used to improve load prediction accuracy or to tune hyperparameters, and the capacity budget framework of this paper can serve as a constraint layer for learning-based methods.

## 3. System Model and Research Motivation

### 3.1. System Model

This paper studies a distributed, centerless, ad hoc network scenario consisting of *N* homogeneous terminal nodes with single-hop fully connected topology. The nodes operate in half-duplex mode, where transmission and reception cannot occur simultaneously. All nodes are equipped with the improved SPMA link-layer protocol proposed in this paper; all modules included in the protocol are completed independently locally, requiring no inter-node signaling exchange of load information. The physical layer adopts a time-division pulse transmission mechanism, where data packets are encoded and split into multiple pulse groups for transmission. The receiving end can complete packet decoding upon receiving half or more of the pulses. Each node’s priority queue buffer is finite, and packets that cannot enter the queue are directly discarded. Each node generates *M* priority services, where priority *S*_0_ is the highest priority and priority *S_M_*_−1_ is the lowest. The typical traffic of *S*_0_ consists of delay-sensitive collection and control data, which have mandatory target transmission success rate requirements and are the core guaranteed services of the system; priorities *S*_1_ through *S_M_*_−1_ are large-capacity, delay-tolerant services without mandatory transmission success rate requirements, but should be given as many transmission opportunities as possible when the channel is idle. It is assumed that the packet arrivals of each priority service at each node are mutually independent and follow Poisson processes.

### 3.2. Research Motivation

The main research motivation of this paper is the discovery that the existing SPMA protocol, which relies solely on COS-versus-threshold comparison for scheduling decisions, is highly susceptible to instantaneous COS fluctuations. This leads to massive accumulation of packets in priority queues, which are then transmitted intensively over short time periods, causing severe collisions among packets from different nodes and different queues. In many cases, the packet transmission success rate is even lower than that of the simple ALOHA protocol without any threshold restrictions.

**Figure 2:**
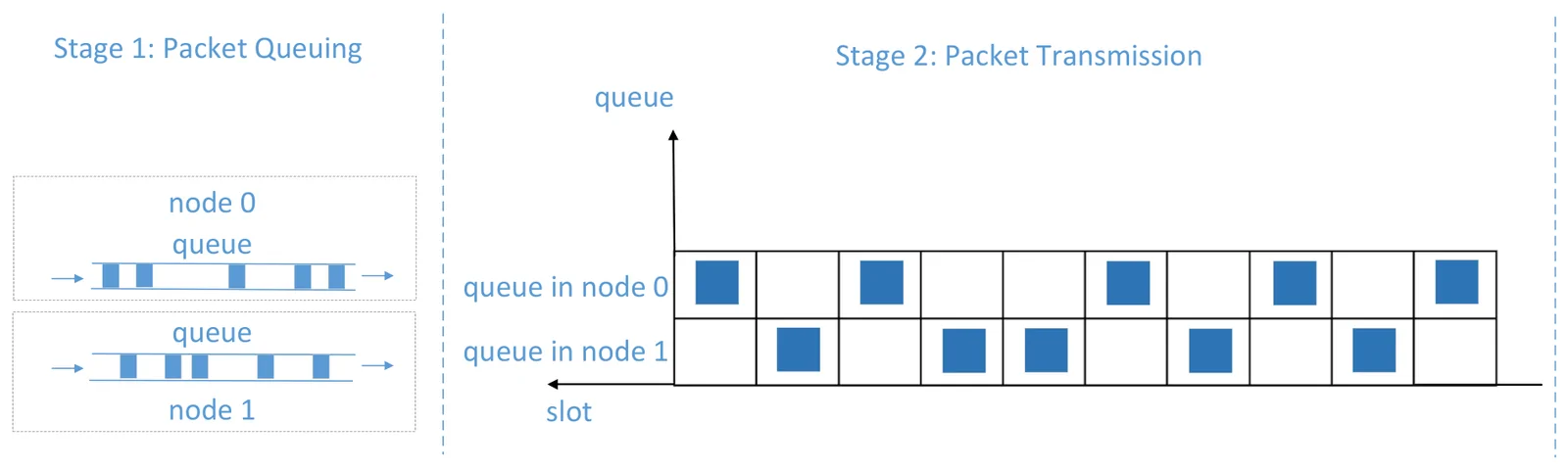
Packet queueing and scheduling mechanism of the ALOHA protocol. Note: The time-slot index increases toward the left side; packets located on the right side are transmitted earlier.

In the ALOHA protocol, since packets are transmitted upon arrival, whether packets sent to the channel by different nodes collide is entirely determined by the packet arrival process. Typically, the packet arrival processes of different nodes are mutually independent, and the arrival process within each node is relatively random. This randomness results in a relatively low collision probability. As shown in Figure 2, after packets are sent to the channel, due to the randomness of the arrival process, no packets collide. Assuming that the packet arrival process at each node follows mutually independent Poisson processes, the process by which packets arrive at the channel under the ALOHA protocol also follows independent Poisson processes. In this case, the packet transmission success rate is exactly the same as Formula (1) introduced earlier. For example, assuming the number of nodes *N* = 2, the number of channels *N_f_* = 1, the number of pulses per packet *N_b_* = 1, and the window length *W* = 200 time slots, then *P_s_* = exp(−*COS*/200). If *COS* = 20, i.e., an average of 10 packets arrive at each node every 200 time slots, the packet transmission success rate *P_s_* can reach 90.48%, while if *COS* = 2, i.e., an average of 1 packet arrives at each node every 2000 time slots, *P_s_* can reach 99.0%.

**Figure 3:**
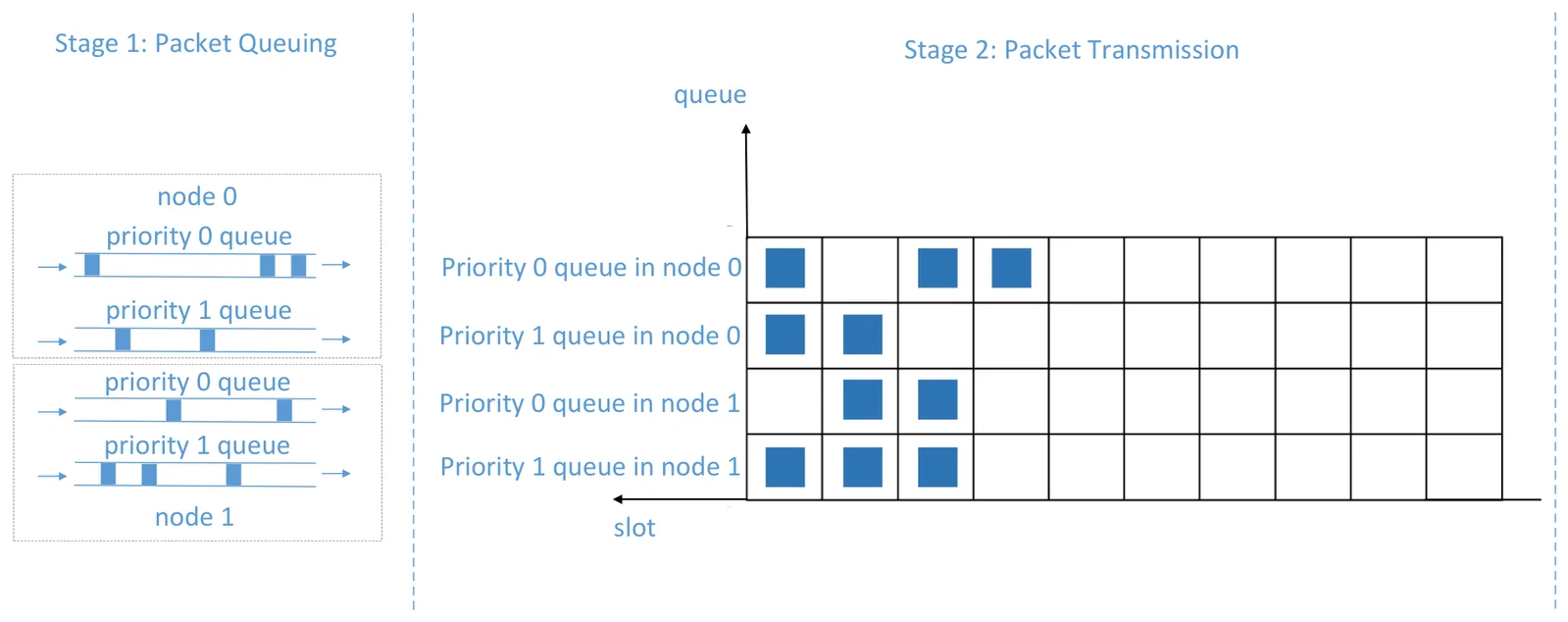
Packet queueing and scheduling mechanism of the traditional SPMA protocol. Note: The time-slot index increases toward the left side; packets located on the right side are transmitted earlier.

In fact, reviewing Section 2.1, one finds that whether using analytical or measurement-based methods, the determination of SPMA thresholds assumes that the packet arrival process at the channel follows the same independent Poisson distribution as the ALOHA protocol. However, this assumption is difficult to satisfy in SPMA networks (unless the total generated traffic of the entire network is far less than the channel load): whether a packet can be sent to the channel directly depends on the comparison between COS and the threshold. Since COS as a real-time measurement is continuously varying, when COS exceeds the threshold, packets in the corresponding queues accumulate. Once COS falls below the threshold, packets are sent intensively in a short time, causing severe collisions (as shown in Figure 3, where 9 out of 10 packets collide). Although SPMA employs a random backoff mechanism to alleviate concentrated packet transmission, once packet accumulation occurs, the mutually independent random arrival intervals no longer exist. At this point, the additional randomness that the backoff mechanism can introduce is extremely limited, and collisions become difficult to avoid [10,15,31].

The above is a qualitative description of the research motivation. Below, through four simulation scenarios, the causes of the above problems and their adverse effects are specifically and quantitatively revealed.

The four scenarios are not divided by load level or topology, but are designed following the principle of progressive mechanism introduction: each scenario adds one mechanism factor on the basis of the previous one, so that the causes at three levels—the statistical characteristics of the shared channel itself, the inherent defects of the post-hoc threshold mechanism, and the resource contention under multi-priority coupling—are separated layer by layer. This design follows the control-variable method, so that each phenomenon corresponds to a definite cause. Scenario 1 (ALOHA, single priority, full load) retains only the most basic condition of multiple nodes arriving randomly on a shared channel and establishes the baseline. Scenario 2 (SPMA, single priority, full load) introduces the threshold decision and random backoff of SPMA on the basis of Scenario 1, and is used to examine the effect of the threshold mechanism itself. Scenario 3 (SPMA, two priorities, each with full load, total overload) introduces the second-highest priority service on the basis of Scenario 2, and is used to examine the resource contention under multi-priority coupling plus total overload. Scenario 4 (SPMA, two priorities, each with half load, total full load) increases the average arrival interval of both priorities on the basis of Scenario 3, and is used to examine the case where the total load does not exceed the channel capacity but the traffic structure changes.

**Scenario 1: Using the ALOHA protocol, only *S*_0_ priority**

**Figure 4:**
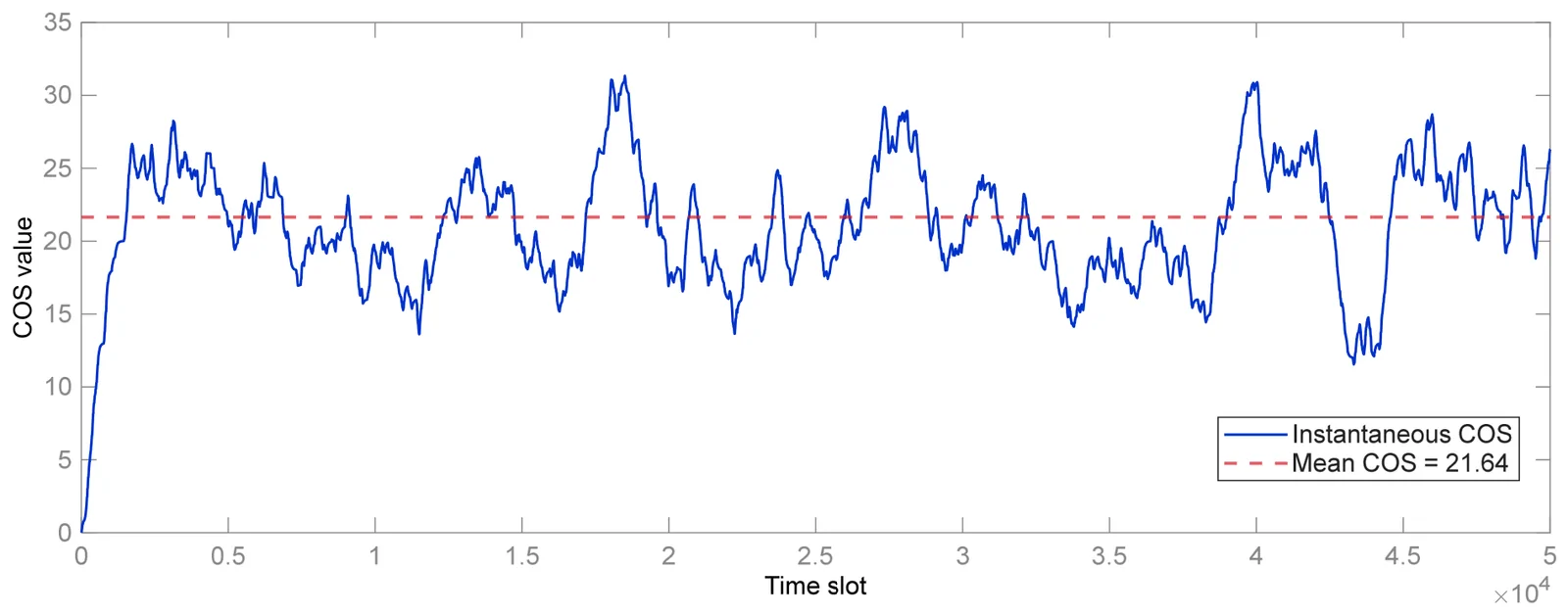
COS curves for Scenario 1.

According to the measurement-based baseline threshold determination method, an eight-node, single-channel, fully connected network is constructed. The network has only the highest priority *S*_0_ service. The MAC layer of each node uses the ALOHA protocol. The initial average arrival interval of the service is *T*_0_ = 200 time slots. Under this interval, the simulation runs for 200,000 time slots. During this period, using a sliding detection window length of 200 time slots, the instantaneous COS value is counted at each time slot. After 200,000 time slots, the mean COS is calculated and the transmission success rate is counted. If the transmission success rate is greater than or equal to the target transmission success rate of 90%, *T*_0_ is gradually decreased in steps of four time slots (i.e., the traffic load *TR* is increased) until the transmission success rate falls below 90%. The previous *T*_0_ value is the minimum arrival interval *T_min_* that guarantees the target transmission success rate, and the corresponding traffic load is the current reference traffic load *TR_base_*. According to the above method, *T_min_* = 76 time slots is obtained. Under this interval, the mean COS is 21.64, i.e., the *COS_base_* that guarantees the target transmission success rate. Figure 4 plots the instantaneous COS values and the mean COS when *T_min_* = 76 time slots. This measured calibrated value of 21.64 is slightly higher than the theoretical baseline occupancy rate of 21.07 calculated by Formula (2) (*W* = 200, *N_f_* = 1, *N_b_* = 1, *P_succ_* = 0.9). The deviation arises from the random fluctuation of Poisson arrivals within the finite statistical window and the quantization granularity of 4-slot steps, which is normal calibration error. The following sections all use this measured value of 21.64 as the highest-priority threshold.

**Scenario 2: Using the SPMA protocol, only *S*_0_ priority, full traffic load**

**Figure 5:**
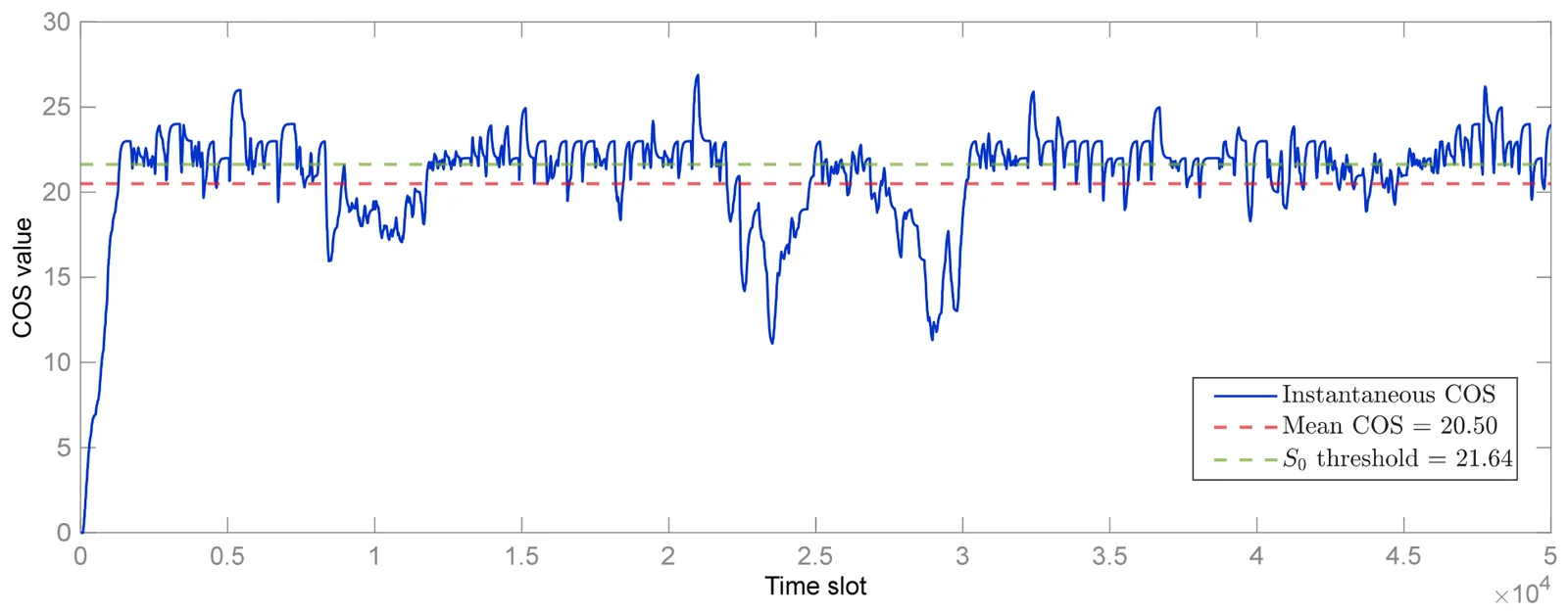
COS curves for Scenario 2.

Based on the test results of Scenario 1, the ALOHA protocol is modified to the SPMA protocol. The threshold for *S*_0_ is set to *COS_base_* = 21.64, and each node has full traffic load (the average arrival interval remains *T_min_* = 76 time slots). Re-running the simulation reveals that the transmission success rate is only 58.46%, far below the 90% target. The reason can be seen from the COS curves in Figure 5: 47.59% of the instantaneous COS values exceed *COS_base_*, meaning that in 47.59% of the time slots, transmission is not allowed because the instantaneous COS exceeds the threshold. The transmission opportunities for all nodes are restricted to the remaining 52.41% of time slots, causing each node’s transmissions to be too concentrated and collisions to intensify. In contrast, since ALOHA packets are transmitted upon arrival and the transmission times are dispersed, there are fewer collisions. Thus, the threshold restriction of SPMA causes multi-node transmissions to become overly concentrated, which is the fundamental reason why the transmission success rate is significantly lower than that of the ALOHA protocol.

**Scenario 3: Using the SPMA protocol, with both *S*_0_ and *S*_1_ priorities coexisting, each with full traffic load, and total load being overloaded**

**Figure 6:**
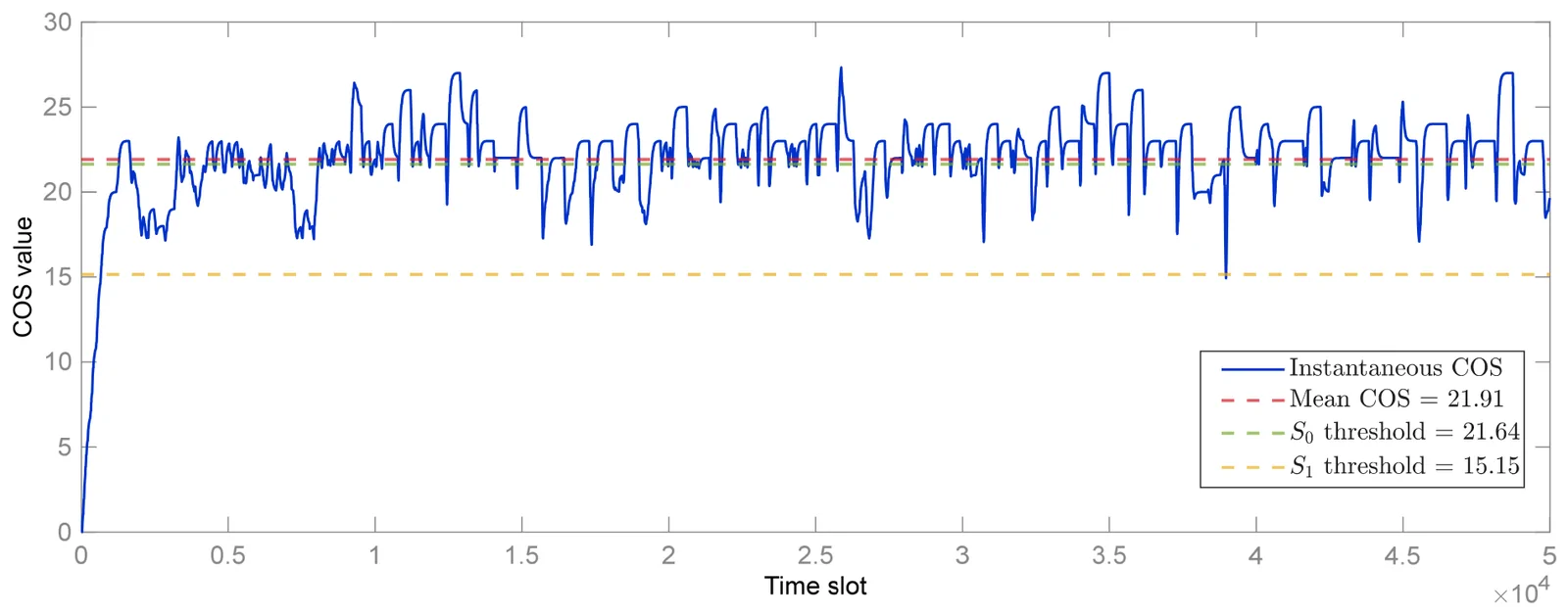
COS curves for Scenario 3.

We keep the *S*_0_ traffic of all nodes at full load while additionally adding the second-highest priority service *S*_1_ (also at full load, with the threshold set to 0.7 times the *S*_0_ threshold, i.e., 15.15). According to the SPMA scheduling decision mechanism, the newly added *S*_1_ should not affect the transmission success rate of *S*_0_, but the measured transmission success rate of *S*_0_ drops sharply from 58.46% to 9.35%. As shown in Figure 6, although the total traffic in this scenario is twice that of Scenario 2, the mean COS of 21.91 is not much different from the 20.50 of Scenario 2, indicating that the threshold decision does indeed limit the total number of packets sent to the channel. However, counting the actual number of packets sent by the two priorities reveals that the ratio of *S*_1_ packets to *S*_0_ packets sent to the channel is 55.52%. That is, even though the *S*_1_ threshold is much lower than the *S*_0_ threshold, *S*_1_ can still exploit the extremely limited transmission opportunities (only 0.56% of instantaneous values are below the *S*_1_ threshold) brought about by COS curve variations, sending packets that have been accumulated over a long time in a concentrated burst. Meanwhile, since 65.67% of instantaneous values are above the *S*_0_ threshold, the accumulation of *S*_0_ packets is further aggravated compared to Scenario 2. These factors cause *S*_0_ packets to collide simultaneously with *S*_0_ and *S*_1_ packets from other nodes, further reducing the transmission success rate. This result demonstrates that due to the time-varying nature of COS, even if the low-priority threshold is lower than the high-priority threshold, it still has opportunities to transmit, easily causing the core design objective of the SPMA protocol to fail: it cannot provide sufficient protection for high-priority traffic.

**Scenario 4: Using the SPMA protocol, with both *S*_0_ and *S*_1_ priorities coexisting, each with light traffic load, and total load being full load**

**Figure 7:**
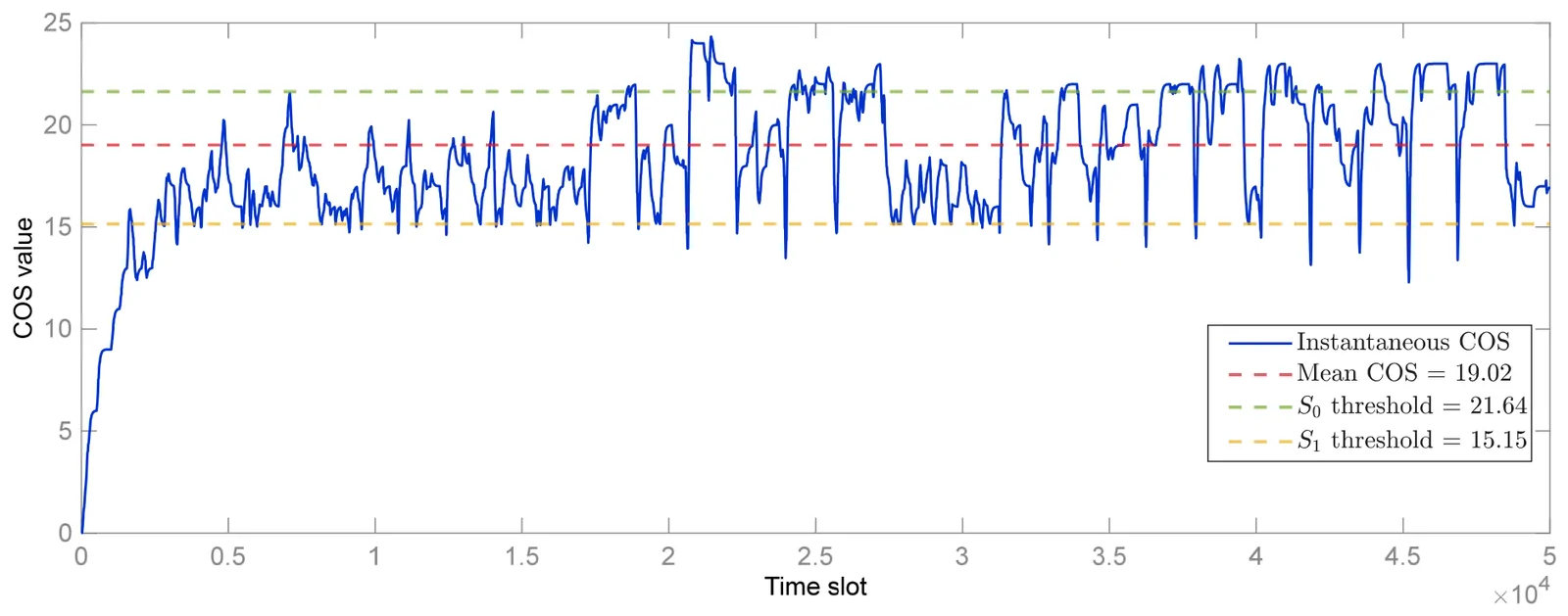
COS curves for Scenario 4.

We increase the average arrival interval of both *S*_0_ and *S*_1_ services at all nodes to 152 time slots, i.e., each service load is only half of the reference traffic load *TR_base_*, and the total equals *TR_base_*, with other parameters the same as Scenario 3. The measured transmission success rates of *S*_0_ and *S*_1_ are 81.13% and 13.03%, respectively. In Scenario 1, even when the *S*_0_ service was at full load, its transmission success rate was still as high as 90%. This indicates that even in scenarios where the *S*_0_ traffic load is much lower than in the case using the ALOHA protocol, the performance of the SPMA protocol is still far inferior to that of the ALOHA protocol, and the underlying reasons are essentially the same as in Scenario 3 (see Figure 7).

Below, the experimental results and their causes from the above four scenarios are summarized in Table 1.

In summary, the post-hoc threshold interception mechanism of the traditional SPMA protocol has inherent defects, easily causing packet accumulation and aggregated collisions, significantly deteriorating the transmission success rates of multi-priority services. To address this problem, this paper steps outside the existing control framework of the SPMA protocol and proposes DW-PA-SPMA, a protocol based on long and short dual-window differentiated priority load statistics and source-end probabilistic pre-admission. Traffic shaping is performed at the packet generation source, and safe bandwidth is hierarchically reserved for high-priority services, aiming to provide effective differentiated service guarantees for multi-priority services.

## 4. Dual-Window Probabilistic Admission SPMA Protocol

### 4.1. Basic Idea

The traditional SPMA protocol adopts a post-hoc threshold–random backoff traffic management architecture: after packets are enqueued, at each transmission time slot, the real-time channel occupancy statistic COS is compared with the priority threshold, and packets that do not meet the condition remain in the queue and perform random backoff retries. As described in Section 3.2, this mechanism has two inherent structural defects. First, the management position is post-hoc; when overload occurs, packets are not limited at the source but instead accumulate massively in queues of each priority. Once the instantaneous channel occupancy falls below the threshold, the accumulated packets from multiple nodes across the network are sent in concentrated bursts, causing aggregated collisions. This makes the actual channel packet transmission sequence deviate from the Poisson arrival assumption on which SPMA theoretical analysis relies, resulting in severe degradation of transmission success rate. Second, the threshold–backoff mechanism has strong memory effects on the adjustment of packet transmission timing. The originally independent and random traffic flow is transformed into bursty traffic, and the analytical relationship between threshold and success rate derived based on the Poisson model no longer holds, making system performance difficult to analyze and predict.

To address these defects, this paper constructs an improved approach based on the mathematical property of independent thinning of Poisson processes: under the condition that mutually independent Bernoulli probability sampling is performed between each packet, if the original packet stream follows a Poisson process, the packet stream after probabilistic admission filtering with probability *p* still follows a Poisson distribution, with the arrival rate scaled proportionally to *p* times the original rate. Based on this property, this paper shifts traffic management from the post-hoc decision at the transmission time slot to before packet generation and enqueuing, introducing source-end probabilistic admission control. For each newly generated priority-*i* packet, an independent decision is made according to the real-time computed admission probability *p_i_* to determine whether it is allowed to enter the MAC layer transmission queue; packets that do not pass admission are directly discarded, without entering the queue or triggering backoff.

Source-end probabilistic admission brings three essential changes:

(1) **Preserving Poisson statistical characteristics:** Source-end independent probabilistic dropping is a memoryless thinning transformation of the Poisson process. After admission shaping, the traffic stream entering the channel still approximately follows a Poisson process. The transmission success rate analytical formula derived in the classic SPMA literature [11] based on the Poisson assumption remains valid, and system performance is analytically tractable.
(2) **Eliminating queue packet accumulation:** Overload traffic is probabilistically truncated at the traffic source. The queue only stores packets that have already been admitted, fundamentally eliminating the large-scale queuing and backoff waiting of packets. The end-to-end delay can be compressed to the time-slot level.
(3) **Achieving quantifiable channel capacity budgeting:** Combining differentiated priority statistics to obtain the long-term steady-state channel occupancy, the admission probability at each level is dynamically computed according to the principle of “highest priority guaranteed first, lower priorities reuse the remaining channel capacity,” clamping the overall long-term average load of the entire network within the baseline channel occupancy rate, ensuring that high-priority services stably meet the target transmission success rate across a wide load range from light load to heavy load.

For the above three aspects, further clarification is needed. First, the source-end probabilistic admission of this paper is fundamentally different from the post-hoc threshold interception of traditional SPMA in terms of the traffic management paradigm: the post-hoc scheme makes decisions at the transmission time slot, when packets have already entered the queue, occupied buffers, and participated in channel contention, so that packets dropped under overload due to a full queue or waiting timeout have already consumed queue buffers and undergone multiple random backoffs; the proposed scheme makes decisions at the instant of packet generation, so that packets that are not admitted neither enter the queue nor occupy buffers or channel contention resources, and the dropping is controlled by the admission probability computed layer by layer according to priority by the capacity budget module, which is a quantitative dropping whose statistical consequences are theoretically computable and predictable. Second, the invariance of Bernoulli thinning holds strictly only when the input stream itself is an independent Poisson stream. When the input stream consists of bursty, strongly correlated, or periodic traffic, the output stream after admission no longer strictly maintains a Poisson distribution; nevertheless, source-end admission can still constrain the total traffic injected into the channel within the capacity budget and suppress queue accumulation. This effect is guaranteed by the computational structure of the capacity budget and is independent of the specific form of the arrival distribution, as further discussed in Section 6.2.

### 4.2. Protocol Design

**Figure 8:**
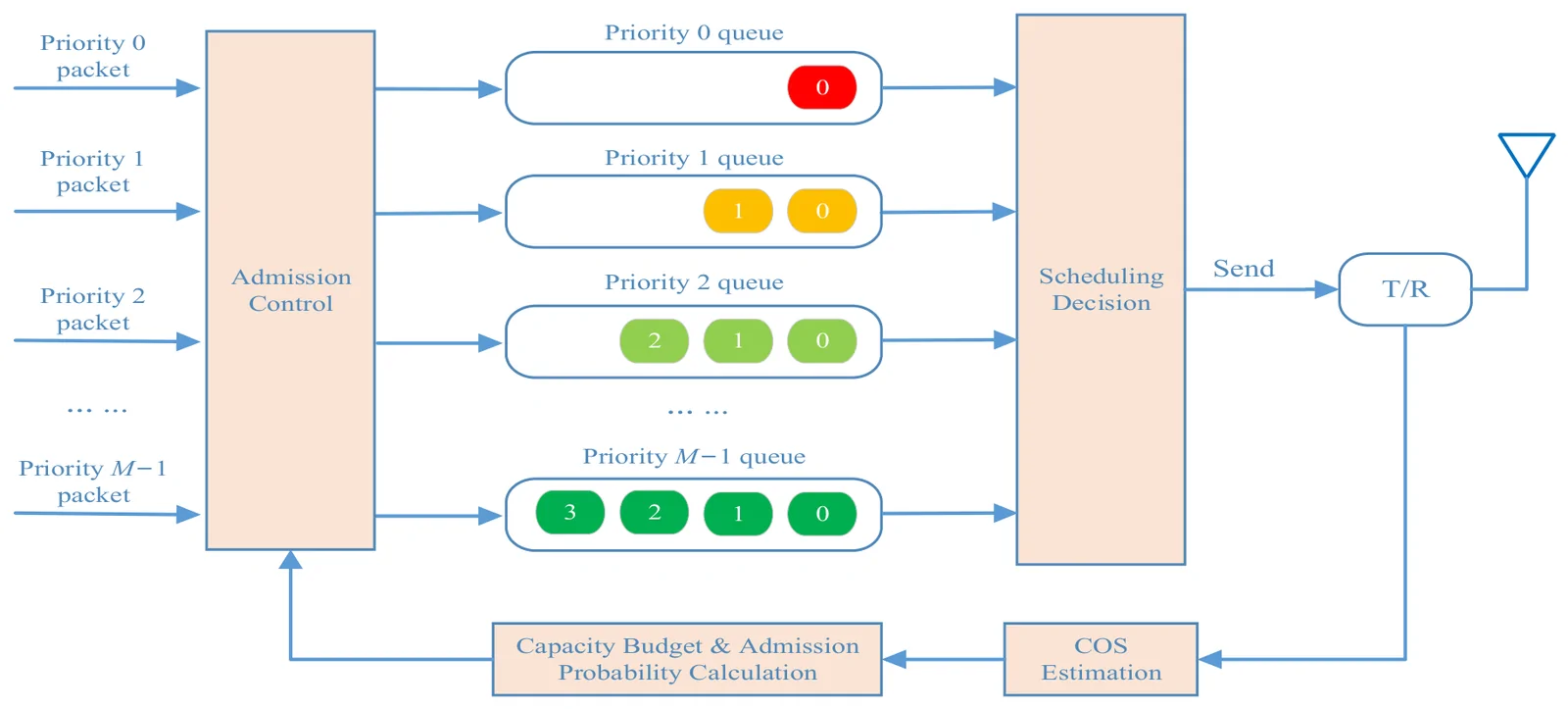
Overall framework of the DW-PA-SPMA protocol.

To implement source-end probabilistic admission, the DW-PA-SPMA protocol architecture consists of four main modules: the COS estimation module, the capacity budget and admission probability computation module, the admission control module, and the scheduling decision module. The COS estimation module adopts a long–short dual-window architecture, where the short window is responsible for capturing instantaneous channel occupancy, and the long window is used to extract the steady-state load after filtering out jitter. The capacity budget module takes the baseline channel occupancy rate corresponding to the target transmission success rate as a constraint, hierarchically reserves channel resources, and outputs the admission probability for each priority. The admission control module performs probabilistic gating at the packet generation stage. The scheduling decision module performs strict priority scheduling for already admitted packets without repeating COS threshold verification, achieving “admission means transmission.” The complete protocol architecture is shown in Figure 8.

The specific functions completed by each module are as follows.

**(1) COS Estimation Module**

This module independently counts both instantaneous and long-term channel occupancy for each priority:

The short window *W_s_* independently counts the instantaneous channel occupancy *COS_i_*(*t*), (*i* = 0, 1, …, *M* − 1) for each priority, i.e., the number of *S_i_* packets sent on the channel in the most recent *W_s_* time slots:

(3) $${\mathrm{COS}}_{i}(t)=\sum_{k=t-W_{s}+1}^{t}X_{i}\left(k\right),$$

where *X_i_*(*k*) ≥ 0 is the number of *S_i_* packets sent on the channel in time slot *k*.

The long window *W_L_* independently counts the long-term steady-state channel occupancy ${\bar{\mathrm{COS}}}_{i}$ for each priority, using exponential weighted moving average (EWMA) to eliminate instantaneous load jitter:

(4) $${\bar{\mathrm{COS}}}_{i}\left(t\right)=\left(1-\alpha_{L}\right)\cdot \;{\bar{\mathrm{COS}}}_{i}\left(t-1\right)+\alpha_{L}\cdot {\mathrm{COS}}_{i}^{‘}\left(t\right),$$

where *α_L_* is the long-window exponential smoothing coefficient, with default value *α_L_* = 1/*W_L_*, equivalent to a weighted average of the load over the past *W_L_* time slots; ${\mathrm{COS}}_{i}^{‘}\left(t\right)$ is the short-window count *COS_i_*(*t*) after a scaling transformation before entering the EWMA recursion: ${\mathrm{COS}}_{i}^{‘}\left(t\right)={\mathrm{COS}}_{i}\left(t\right)\cdot W_{L}/W_{S}$, converting the short-window statistic to the long-window statistical scale.

**(2) Capacity Budget and Admission Probability Computation Module**

The core function of this module is to dynamically compute a “source-end admission probability” *p_i_* for each priority, based on the long-term steady-state channel occupancy ${\bar{\mathrm{COS}}}_{i}$ for each priority provided by the COS estimation module, combined with the baseline channel occupancy rate *COS_base_* corresponding to the target transmission success rate. The computation follows the hierarchical reservation principle of “highest priority guaranteed first, lower priorities share the remaining capacity.”

First, the average arrival interval *T_i_* (in time slots) of *S_i_* is converted into the channel capacity demand *D_i_* within the long window *W_L_*:

(5) $$D_{i}=\frac{N}{T_{i}}\cdot W_{L},\;i=0,\;1,\;\ldots ,\;M-1,$$

where *N*/*T_i_* is the average packet generation rate of *S_i_* packets across all nodes in the network (packets/time slot). Multiplying this value by the long window length *W_L_* yields the total packet demand *D_i_* of the *S_i_* service within the long window.

Subsequently, the available channel capacity *C_i_* for *S_i_* is defined as:

(6) $$\left\{\begin{array}{l} C_{0}={\mathrm{COS}}_{\mathrm{base}}^{‘}\\ C_{i}={\mathrm{COS}}_{\mathrm{base}}^{‘}-\sum_{j=0}^{i-1}{\bar{\mathrm{COS}}}_{j},\;i=1,\;\ldots ,\;M-1, \end{array}\right.$$

where ${\mathrm{COS}}_{\mathrm{base}}^{‘}$ is the baseline channel occupancy rate *COS_base_* counted under the short window converted to the long window scale, i.e., ${\mathrm{COS}}_{\mathrm{base}}^{‘}={\mathrm{COS}}_{\mathrm{base}}\cdot \frac{W_{L}}{W_{S}}$. The above formula indicates that the available channel capacity *C*_0_ for the highest priority *S*_0_ is directly equal to ${\mathrm{COS}}_{\mathrm{base}}^{‘}$, while the available capacity *C_i_* for lower priority *S_i_* (*I* ≥ 1) is the remainder after deducting the long-term occupancy of all higher-priority services from ${\mathrm{COS}}_{\mathrm{base}}^{‘}$.

Based on the channel capacity demand *D_i_* and available channel capacity *C_i_* for each priority, the admission probability *p_i_* is analytically computed as follows:

(7) p0=min 1,C0D0pi=max 0,min1,CiDi, i=1, …,  M−1.

Formula (7) gives clear traffic shaping instructions: when the demand *D_i_* of a certain priority service does not exceed its available capacity *C_i_*, *p_i_* = 1, indicating that all packets are allowed to enter the queue; when the demand exceeds the available capacity, *p_i_* = *C_i_*/*D_i_* < 1, indicating that newly generated packets are allowed to enter the queue only with probability *p_i_*, and the excess is probabilistically rejected at the source. This mechanism transforms the passive congestion control of “enqueue then compete and backoff if failed” in traditional protocols into the preventive congestion avoidance strategy of “active flow limiting according to capacity budget before enqueuing.”

Finally, the updated long-window smoothed load ${\bar{\mathrm{COS}}}_{i}$ is fed back to this module at each time slot, forming a closed-loop regulation that enables the admission probability *p_i_* to closely follow the trend of traffic load variations.

**(3) Admission Control Module**

This module is the final execution unit for implementing source-end traffic shaping. It inserts a probabilistic gating step before the upper-layer service submits generated packets to the MAC layer. For each newly generated *S_i_* packet, the module performs the following operations:

- Obtains the current time-slot admission probability *p_i_* computed by the capacity budget and admission probability computation module;
- Generates an independent random number *u* uniformly distributed in *U*(0,1);
- Performs the gating decision: if *u* ≤ *p_i_*, the packet is allowed to enter the corresponding priority queue to wait for transmission; conversely, if *u* > *p_i_*, the packet is directly discarded without triggering any MAC-layer retransmission or backoff mechanism.

The core advantage of this design is that it shifts the negative effects of network overload (collisions, queuing delay, buffer overflow) from the backend transmission queues and physical channel to the frontend packet generation source. Through probabilistic dropping, the total traffic entering the network is mathematically expected to be strictly controlled within the channel capacity budget, fundamentally preventing network performance collapse caused by traffic overload.

**(4) Scheduling Decision Module**

This module selects packets from the heads of non-empty priority queues at each time slot for transmission to the physical channel. The scheduling policy strictly follows the order from highest to lowest priority: the scheduler first checks the highest priority *S*_0_ queue; if non-empty, it immediately sends the head packet; and otherwise, it sequentially checks the *S*_1_, *S*_2_, …, *S_M_*_−1_ queues.

The key innovation here is that already admitted packets do not require any form of COS threshold comparison or random backoff when scheduled for transmission. This is because the admission control module has already guaranteed from the total volume that the total demand of all enqueued packets does not exceed the channel capacity. Therefore, any additional conditional checks at the scheduling stage are redundant and may introduce unnecessary delay. This “admission means transmission” mechanism is the fundamental reason why the proposed algorithm can stabilize end-to-end delay at an extremely low level.

**Figure 9:**
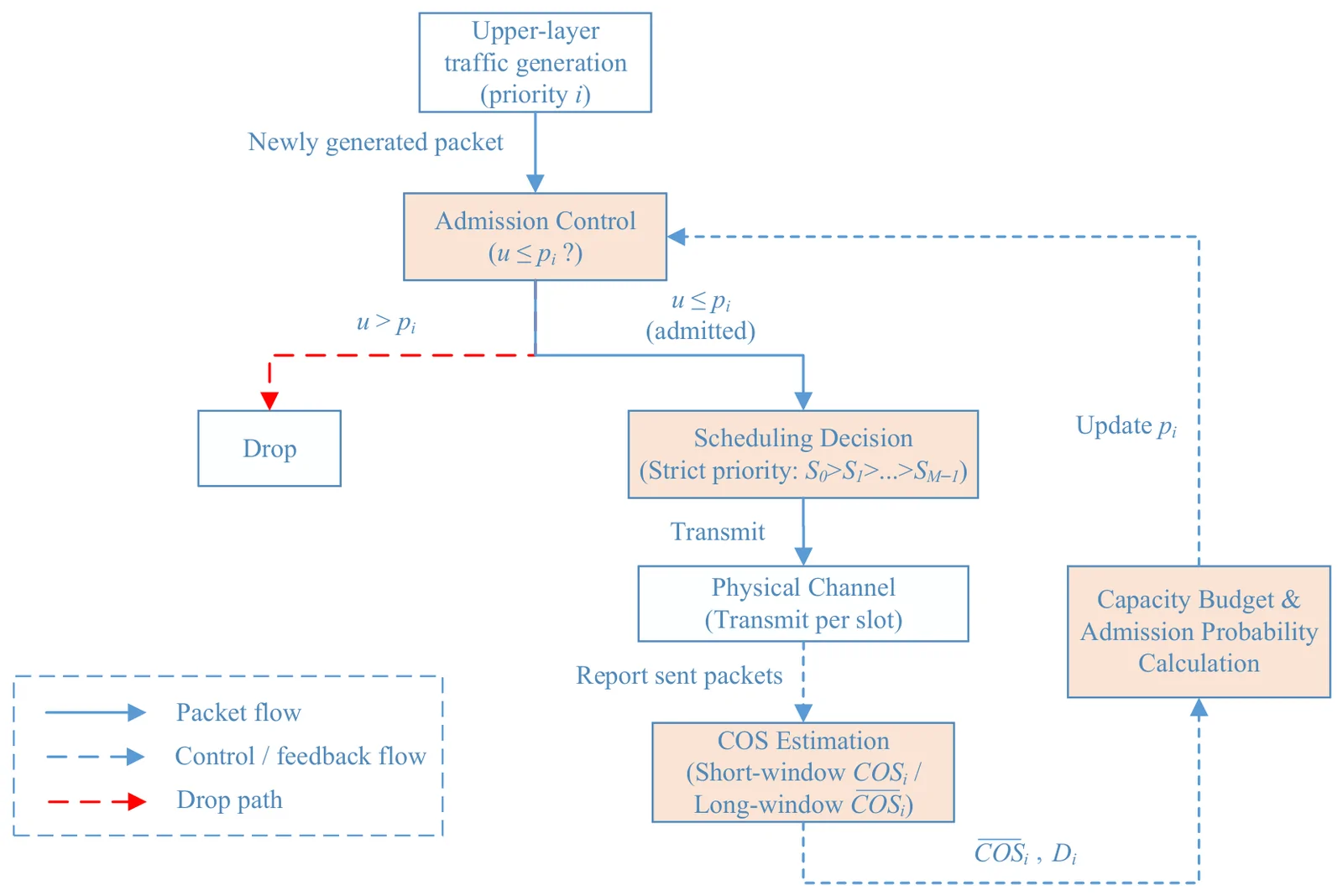
Closed-loop control architecture and signal flow of DW-PA-SPMA.

In summary, the complete data flow relationship among the four modules of the DW-PA-SPMA protocol is shown in Figure 9. In the figure, the solid lines represent the main data path: packets generated by the upper layer first pass through the admission control module, where source-end traffic shaping is performed according to the current time-slot admission probability; packets that pass admission enter the scheduling decision module and are directly sent to the physical channel according to strict priority; the physical channel reports the number of packets actually sent in the current time slot to the COS estimation module. The dashed lines in the figure represent the control feedback path: the COS estimation module counts the short-term instantaneous occupancy and long-window smoothed steady-state load for each priority, and sends the load parameters along with the capacity demand *D_i_* for each priority to the capacity budget and admission probability computation module. The latter computes the available capacity for each priority according to Formula (6) and solves the admission probability at each level according to Formula (7), feeding it back to the admission control module for the traffic shaping decision in the next time slot. The four modules iterate cyclically at each time slot, implementing preventive and precise management of channel resources through advance flow limiting, demonstrating significant performance advantages over the traditional SPMA protocol.

### 4.3. Parameter Settings

This section determines the principles for setting the short window *W_s_*, the long window *W_L_*, and the parameters in their adaptive mechanism in the DW-PA-SPMA protocol. In this protocol architecture, channel occupancy statistics adopt a two-stage cascade filtering structure consisting of a short-window finite impulse response (FIR) moving average and a long-window exponentially weighted moving average (EWMA). The fundamental reason for using dual windows rather than a single window is that a single window must compromise between estimation noise and tracking lag: too short a window leads to excessive statistical noise, causing the admission probability to oscillate at high frequency; too long a window leads to severe lag in responding to load changes, and the admission probability adjustment cannot keep up with traffic variations. The dual-window structure alleviates both problems simultaneously through responsibility decoupling.

#### 4.3.1. Approximate Analytical Model of Two-Stage Filtering

The short window *W_s_* is a finite-length equal-weight FIR moving average filter. For a ramp-changing load signal, its inherent tracking lag is approximately half the window length *W_s_*/2. The long-window EWMA is a first-order infinite impulse response (IIR) low-pass filter, whose equivalent weighted memory time constant is denoted as *τ*. After cascading the two-stage filter, the overall tracking lag of the load estimation module is:

(8) $$\delta =\frac{W_{s}}{2}+\tau .$$

Under the combined effect of the two-stage filtering, the total estimation variance satisfies the approximate relationship:

(9) $$\sigma^{2}\approx \frac{\mathrm{Var}\left(x\right)}{2{\cdot W}_{s}{\cdot W}_{L}},$$

where *Var*(*x*) is the variance of the original time-slot count. The derivation of Equations (8) and (9) assumes: (1) the input sequence is approximately an independent and identically distributed Poisson count, whose variance equals its mean; (2) the short-window equal-weight averaging compresses the original variance to *Var*(*x*)/*W_s_*; (3) the steady-state variance gain of the long-window EWMA on the already smoothed input is approximately 1/(2*W_L_*); (4) the time scales of the two-stage filtering are sufficiently separated, i.e., *W_s_* ≪ *W_L_*. The above formulas are approximate analytical results, mainly used for qualitatively understanding the trend of system performance variation with parameters.

Based on the above cascade model, the responsibilities of the two types of windows are clearly separated: the short window *W_s_* is a static statistical aggregation parameter, responsible for denoising the original discrete time-slot counts and providing a noise-controllable input signal for the EWMA, without determining the dynamic tracking performance of the system; the long window *W_L_* is a dynamic tracking parameter, serving as the core degree of freedom controlling the system’s response speed to load changes, and its value needs to match the time scale of traffic load variations.

#### 4.3.2. Equivalent Relationship Between EWMA Smoothing Coefficient and Long Window

The long window adopts the EWMA iterative update of steady-state load as shown in Equation (4). To analyze its steady-state variance characteristics, consider a general first-order IIR filter. Let its input be a stationary random sequence with variance *Var*(*x*), and let the variance of the output ${\bar{\mathrm{COS}}}_{i}\left(t\right)$ be *Var*(*y*). Under steady-state conditions, the following holds:

(10) $$\mathrm{Var}(y)=(1-\alpha_{L}{)}^{2}\;\cdot \mathrm{Var}(y)+\alpha_{L}^{2}\;\cdot \mathrm{Var}(x).$$

In the actual operation of the protocol, this input sequence corresponds to the scaled ${\mathrm{COS}}_{i}^{‘}\left(t\right)$ in Equation (4), whose variance already includes the scaling factor ${\left(W_{L}/W_{S}\right)}^{2}$. The derivation of Equations (10)–(13) holds generally for this input sequence, and the equivalent relationship between *W_L_* and *α_L_* remains valid.

Thus, the output variance expression is:

(11) $$\mathrm{Var}(y)\;=\;\frac{\alpha_{L}}{2-\alpha_{L}}\cdot \mathrm{Var}(x).$$

The equivalent weighted memory time constant of the EWMA filter is defined as the weighted average of historical input times according to EWMA weights:

(12) $$\tau \;=\;\sum_{k=0}^{\infty }k\cdot \alpha_{L}{\left(1-\alpha_{L}\right)}^{k}=\frac{1-\alpha_{L}}{\alpha_{L}}.$$

When *α_L_* ≪ 1, we have *τ* ≈ 1/*α_L_*. According to the time constant equivalence criterion, letting *τ* ≈ *W_L_* yields:

(13) $$\alpha_{L}\approx \frac{1}{W_{L}}.$$

#### 4.3.3. Constraints and Selection of the Short Window *W_s_*

The selection of the short window *W_s_* must simultaneously satisfy two constraints.

**(1) Statistical aggregation and denoising constraint.** If *W_s_* is too small, the short-window output will degenerate into time-slot-level 0–1 discrete occupancy counts, and the available capacity *C_i_* for low priorities will oscillate violently near zero, causing the low-priority admission probability to hop between 0 and 1 at high frequency, manifesting macroscopically as low-priority service “starvation.” Source-end probabilistic admission preserves the Poisson property of traffic arrivals, and the aggregated count within the window approximately follows a Poisson distribution with expectation *μ* = *N*·*W_s_*/*T*_0_, where *N* is the number of nodes and *T*_0_ is the average arrival interval of the highest-priority service. The validity of the Poisson approximation requires *μ* ≥ 5. Under full-load conditions (*T*_0_ = 76 time slots, *N* = 8), we have *μ* ≈ 0.1053·*W_s_*, giving *W_s_* ≥ 48 time slots. The simulation results in Section 5.2.1 verify the effectiveness of this lower bound: when *W_s_* ≤ 10, low-priority services experience starvation; when *W_s_* ≥ 50, normal operation is restored.

**(2) Dynamic lag decoupling constraint.** It is required that *W_s_* ≪ 2*W_L_*, ensuring that the half-window lag introduced by the short window accounts for a relatively small proportion of the total lag, and that the system’s dynamic tracking performance is dominated by the long-window EWMA.

Among the above two constraints, the statistical aggregation and denoising constraint is a hard constraint that cannot be breached; the dynamic lag decoupling constraint is a performance optimization constraint, and moderate relaxation will weaken the decoupling effect of the dual windows but the protocol can still function normally. This paper takes *W_s_* = 200 time slots, which can satisfy both constraints simultaneously. The sensitivity analysis in Section 5.2.1 shows that for *W_s_* ≥ 50, the worst-case transmission success rate of *S*_0_ across the five segments remains in the flat range of 0.904–0.909. At *W_s_* = 200, the performance is sufficient and remains decoupled from *W_L_*.

#### 4.3.4. Feasible Range of the Long Window *W_L_*

The selection of the long window *W_L_* requires a trade-off between statistical accuracy and tracking capability.

**(1) Theoretical reference for statistical accuracy.** The steady-state output variance of the long-window EWMA is:

(14) $$\mathrm{Var}({\bar{\mathrm{COS}}}_{i})\;=\;\frac{\alpha_{L}}{2-\alpha_{L}}\cdot \rho \cdot (1-\rho ),$$

where *ρ* = *COS_i_*/*W_s_* is the normalized channel occupancy rate based on the short-window statistical scale. Since the actual input has already been denoised by short-window aggregation, the above formula uses the single-slot Bernoulli variance *ρ*∙(1 − *ρ*) as a conservative upper bound for the input variance, and the resulting estimate is a conservatively biased theoretical reference. If the estimated standard deviation is required not to exceed *ε* = 0.01, under typical parameters (*ρ* = 0.1054), solving gives *W_L_* ≥ 472. It should be noted that this lower bound is a theoretical reference value based on the conservative input variance assumption; if calculated according to the actual cascade variance in Equation (9), the statistical lower bound is only on the order of a few time slots. Therefore, this reference value indicates a feasible direction for noise suppression, but the actual selection of *W_L_* is mainly determined by the tracking deviation constraint described below.

**(2) Tracking deviation constraint and scaling law.** Define *RAMP* as the duration of a linear traffic load change (in time slots), characterizing the time scale of load variation. When the load changes linearly at rate Δ/*RAMP* (Δ is the ramp change magnitude of the load), the lag effect of the EWMA filter causes the estimate to deviate by approximately *W_L_*∙Δ/*RAMP*. To ensure this deviation does not exceed an allowable proportion *γ* of the load change magnitude Δ, the following must be satisfied:

(15) $$\frac{\Delta }{\mathrm{RAMP}}\cdot W_{L}\leq \gamma \cdot \Delta .$$

That is:

*W_L_* ≤ *γ* ⋅ *RAMP*. (16)

Between statistical accuracy and tracking capability, the optimal long window takes the feasible upper bound:

(17) $$W_{L}^{*}=\gamma \cdot \mathrm{RAMP}.$$

The coefficient *γ* characterizes the tracking deviation tolerance of the system. Its calibration method is described in Section 5.2.1, with the calibration result *γ*_max_ = 0.2.

#### 4.3.5. Online Estimation of Load Variation Time Scale and Long-Window Adaptation

Equation (17) shows that $W_{L}^{*}$ depends on *RAMP*, which is an inherent property of the traffic environment and cannot be known in advance in actual networks, requiring online estimation. This paper adopts the dual-EWMA difference method to achieve online estimation of *RAMP*.

Two EWMA filters with different time constants are used to track the fast and slow variations in the channel occupancy rate, respectively:

(18) $${\bar{\mathrm{COS}}}_{f}(t)\;=\;(1-\alpha_{f})\cdot {\bar{\mathrm{COS}}}_{f}(t-1)\;+\;\alpha_{f}\cdot {\mathrm{COS}}_{0}\left(t\right),$$

(19) $${\bar{\mathrm{COS}}}_{s}(t)=(1-\alpha_{s})\cdot {\bar{\mathrm{COS}}}_{s}(t-1)\;+\;\alpha_{s}\cdot {\mathrm{COS}}_{0}\left(t\right),$$

where *COS*_0_(*t*) is the short-window statistic of the highest-priority service, and *α_f_* = 1/*τ_f_* and *α_s_* = 1/*τ_s_* are the smoothing coefficients of the fast and slow filters, respectively. The time constants of the fast and slow filters are taken as *τ_f_* = *W_s_* = 200 time slots and $\tau_{s}=W_{L}^{0}=5000$ time slots, respectively, consistent with the time scales of the dual windows, not extra introduced free parameters. The difference *d*(*t*) between the two filter outputs is proportional to the rate of load change:

*d*(*t*) = |*COS_f_*(*t*) − *COS_s_*(*t*)|. (20)

When the load is constant, both filters converge to the same mean, and the expectation of *d*(*t*) is zero; when the load changes linearly with slope *s* = Δ/*RAMP*, the tracking lags of the fast and slow filters are *τ_f_* · *s* and *τ_s_* · *s*, respectively. Under steady state, $d(t)\approx (\tau_{s}-\tau_{f})\cdot s\approx {\;W}_{L}^{0}\cdot s$, i.e., *d*(*t*) is proportional to the load change slope.

Thus, the online estimate of *RAMP* is:

(21) $${\mathrm{RAMP}}_{\mathrm{est}}(t)\;=\;\frac{K}{d(t)},$$

where *K* is a calibration constant. From $\mathrm{RAMP}\approx {\;W}_{L}^{0}\cdot \Delta /d(t)$, we obtain $K\approx W_{L}^{0}\cdot \Delta$. Calibrating with the typical change magnitude of the highest priority from light load (0.5×) to full load (1.0×), i.e., $\Delta \approx {\mathrm{COS}}_{\mathrm{base}}/2\approx 10.8$, gives *K* ≈ 5000 × 10.8 ≈ 5.4 × 10^4^, rounded to 5 × 10^4^ for engineering purposes.

Substituting Equation (21) into Equation (17), the adaptive long window is obtained:

(22) $$W_{L}(t)\;=\;\min\left(W_{L}^{0},\;\gamma_{\max}\cdot {\mathrm{RAMP}}_{\mathrm{est}}\left(t\right)\right),$$

where $W_{L}^{0}$ = 5000 time slots is the design upper bound of the long window, and *γ_max_* = 0.2 is the feasible upper bound calibrated in Section 5.2.1. When the load change accelerates, *d*(*t*) increases and *RAMP_est_*(*t*) decreases, and the long window automatically tightens to ensure tracking capability; when the load is stable, *d*(*t*) approaches zero and *RAMP_est_*(*t*) approaches the upper bound, and the long window returns to the design value to ensure statistical accuracy. In implementation, to prevent *d*(*t*) from approaching zero and causing estimation divergence, *d*(*t*) is clamped to a very small positive value (on the order of 10^−6^ in this paper), and *RAMP_est_*(*t*) is protected by upper and lower bounds (lower bound 5000 time slots, upper bound 10^6^ time slots). Correspondingly, the long-window smoothing coefficient for each priority is adaptively updated as $\alpha_{L}\left(t\right)=1/W_{L}\left(t\right)$.

Based on the analysis in Section 4.3.1, Section 4.3.2, Section 4.3.3, Section 4.3.4 and Section 4.3.5, the parameter values in this paper follow a unified principle. The design upper bound of the long window is jointly constrained by the lower bound of statistical accuracy and the upper bound of tracking capability: the former requires the long window to be long enough to guarantee the stationarity of the load estimate, while the latter requires it to be short enough to follow the variations in the traffic load. The tracking tolerance upper bound is determined by these two bounds, and a margin for the online estimation error is reserved within the performance bound. The short window is jointly determined by the hard lower bound for statistical denoising and the dual-window responsibility decoupling constraint; its value must ensure that the aggregated count within the window satisfies the validity of the Poisson approximation, while being far smaller than the long window to avoid overlapping responsibilities of the two-stage filtering. The calibration constant is used to convert the severity of the load variation into the time scale of the load variation, and is calibrated from the typical magnitude of a load ramp. The time constants of the fast and slow EWMA filters are taken to be of the same order as the short window and the design upper bound of the long window, respectively, so that the time scale of the fast filter matches the instantaneous sensing scale while the slow filter represents the steady-state baseline. The specific values of the above parameters and their sensitivity verification are given in Section 5.2.1.

### 4.4. Algorithm Description

Algorithm 1 describes in detail the complete execution flow of a single node using DW-PA-SPMA in each time slot, integrating the four modules of COS estimation, capacity budget and admission probability computation, admission control, and scheduling decision in sequence. Line 2 first removes timeout head-of-queue packets from each queue; Lines 3–7 correspond to the COS module, updating the short-window instantaneous occupancy *COS_i_* and the long-window smoothed load ${\bar{\mathrm{COS}}}_{i}$, respectively, while converting the arrival interval of each priority into the per-window demand *D_i_*; Lines 8–11 are the online estimation module for the load variation time scale, using the dual-EWMA difference method to estimate *RAMP* in real time and adaptively adjust the long window according to Equation (22); Lines 12–20 are the capacity budget and admission probability computation module, where the highest priority *S*_0_ uses ${\mathrm{COS}}_{\mathrm{base}}^{‘}$ to compute *p*_0_, and lower priorities compute *p_i_* after sequentially deducting the measured occupancy of higher priority ${\bar{\mathrm{COS}}}_{j}$; Lines 21–25 are the admission control module, where newly generated packets pass the random gating with probability *p_i_* and are enqueued, while those that fail are directly discarded; Lines 26–35 are the scheduling decision module, which searches queues in strict priority order from highest to lowest, and admitted packets are sent directly without secondary COS threshold verification, with each node sending at most *N_f_* packets per time slot (subject to the physical channel count constraint).
**Algorithm 1**: Dual-Window Probabilistic Admission SPMA (DW-PA-SPMA)**Input**: Number of nodes *N*, number of priorities *M*, short window *W_s_*, initial long window ${W_{L}}^{0}$, number of channels *N_f_*, baseline channel occupancy rate ${\mathrm{COS}}_{\mathrm{base}}^{‘}$, average arrival interval *T_i_*, scaling law upper bound *γ_max_*, fast and slow filter smoothing coefficients *α_f_*, *α_s_*, calibration constant *K***Output**: Set of packets sent by each node per time slot1:**for** *n* = 1 to *N*
**do**2: Remove timeout head-of-queue packets from each queue of node *n*3:**for** *i* = 0 to *M* − 1 **do**4:        *COS_i_* ← total number of *S_i_* packets on the channel within *W_s_*5:        ${\bar{\mathrm{COS}}}_{i}\leftarrow \left(1-\frac{1}{W_{L}}\right){\bar{\mathrm{COS}}}_{i}+\frac{\mathrm{CO}S_{i}}{W_{s}}$6:        $D_{i}\leftarrow \frac{N}{T_{i}}\cdot W_{L}$7:    **end for**8:     *COS_f_* ← (1 − *α_f_*) · *COS_f_* + *α_f_* · *COS*_0_9:    *COS_s_* ← (1 − *α_s_*) · *COS_s_* + *α_s_* · *COS*_0_10:    ${\mathrm{RAMP}}_{\mathrm{est}}\leftarrow \frac{K}{\left|{\mathrm{COS}}_{f}-{\mathrm{COS}}_{s}\right|}$11:    $W_{L}\leftarrow \min({W_{L}}^{0},\;\gamma_{\max}\cdot {\mathrm{RAMP}}_{\mathrm{est}})$12:    **if** *D*_0_ > 0 **then**
$p_{0}\leftarrow \min\left(1,\frac{{\mathrm{COS}}_{\mathrm{base}}^{‘}}{D_{0}}\right)$13:    **else**
*p*_0_ ← 114:    **end if**15:    **for** *i* = 1 to *M* − 1 **do**16:        $C_{i}\leftarrow {\mathrm{COS}}_{\mathrm{base}}^{‘}-\sum_{j=0}^{i-1}{\bar{\mathrm{COS}}}_{j}$17:        **if** *D_i_* > 0 **then**
$p_{i}\leftarrow \max\left(0,\min\left(1,\frac{C_{i}}{D_{i}}\right)\right)$18:        **else**
*p_i_* ← 119:        **end if**20:    **end for**21:    **for** each newly generated packet *pkt* (priority *i*) **do**22:          **if** *U*(0,1) ≤ *p_i_* **and** queue *i* is not full **then** enqueue *pkt*23:          **else** discard *pkt*24:          **end if**25:    **end for**26:    *sent* ← **0**27:    **for**
*i* = 0 to *M* − 1 **do**28:        **if** queue *i* is non-empty **then**29:            **while**
*sent* < *N_f_* **and** queue *i* is non-empty **then**30:                Send head packet (select a channel randomly from *N_f_*), *sent* ← *sent* + 131:            **end while**32:            **break**33:        **end if**34:**end for**35:**end for**

### 4.5. Comparison with the Traditional SPMA Protocol

To clearly illustrate the fundamental improvements of the proposed algorithm, Table 2 provides a systematic comparison between the traditional SPMA protocol and the DW-PA-SPMA protocol proposed in this paper across seven key dimensions.

## 5. Simulation Verification

### 5.1. Simulation Settings

#### 5.1.1. Network and Traffic Parameters

The simulation is implemented on the MATLAB R2025b platform using a time-slot-driven discrete-event simulation architecture. The simulation parameter values refer to [9,11]. The timeouts of the three priorities *S*_0_, *S*_1_, and *S*_2_ are 2 ms, 10 ms, and 50 ms, respectively, corresponding to the timeliness requirements of the three service types, namely control commands, status reporting, and large-capacity transmission. The simulations in Section 5.2.1 and Section 5.2.2 are conducted in an eight-node, single-channel network, and the simulation in Section 5.2.3 is conducted in a 16-node, four-channel network. The main simulation parameters are shown in Table 3.

The traffic load design for the simulations in Section 5.2.1 consists of five consecutive periods to simulate the gradual process from light load to full load and back. As shown in Table 4, the average arrival interval of the highest priority *S*_0_ sequentially goes through five periods: light load (152 time slots) → load increase (152→76) → full load (76) → load decrease (76→152) → light load (152), completely covering the bidirectional transition process from low to high load and then from high to low. The average arrival intervals of the second-highest priority *S*_1_ and the lowest priority *S*_2_ are 400 time slots and 240 time slots, respectively, and remain fixed across the five periods. The packet arrivals of the three priority services all follow independent Poisson processes.

The performance analysis in Section 5.2.2 adopts a six-segment load scenario to completely cover the gradual process from light load to heavy load and back. As shown in Table 5, the average arrival interval of the highest priority *S*_0_ sequentially goes through six periods: light load (152 time slots) → load increase (152 → 76) → full load (76) → load increase (76 → 38) → heavy load (38) → load decrease (38 → 76). The heavy load segment reaches 2.0 times the full load. The average arrival intervals of the second-highest priority *S*_1_ and the lowest priority *S*_2_ remain 400 time slots and 240 time slots, respectively, fixed across all six periods. Unless otherwise specified, the packet arrivals of the three priority services all follow independent Poisson processes.

The simulation in Section 5.2.3 also adopts a six-segment load scenario to fully cover the various gradual processes of the load in a 16-node, four-channel network. The load variation in the highest priority at each stage is shown in Table 6; the average arrival intervals of the second-highest and lowest priorities are 200 time slots and 120 time slots, respectively, and remain fixed across the six periods. The packet arrivals of the three priority services all follow independent Poisson processes.

#### 5.1.2. Comparison Algorithms

To comprehensively evaluate the performance, two representative static threshold algorithms and one dynamic threshold algorithm are selected as baselines. The highest-priority target success rate for all comparison algorithms is set to 90%.

**Static threshold methods:**

- Uniform-Diff [12]: A classic static threshold scheme. The highest-priority threshold is set to the short-window-scaled *COS_base_*, and the remaining priority thresholds are decreased by a fixed equal difference of 3, i.e., {21.64, 18.64, 15.64}. This scheme is simple to implement but cannot adapt to load changes.
- BiLSTM-SPMA [15]: An improved “time series prediction + fixed threshold” scheme. It uses a bidirectional LSTM network to predict channel load, aiming to improve COS estimation accuracy. However, its thresholds at all priority levels are still statically configured offline, derived by back-calculation according to traffic arrival proportions. Its short-window-scaled thresholds under light load, full load, and heavy load segments are {21.64, 15.75, 10.83}, {21.64, 18.23, 14.43}, and {21.64, 18.55, 16.23}, respectively. It should be noted that this baseline uses offline reconfiguration of static thresholds for each load segment, which is equivalent to knowing the load segment boundaries in advance, a setting that favors its performance.

**Dynamic threshold method:**

- RA-SPMA [19]: A load-feedback-based closed-loop dynamic threshold scheme. It uses the short-window-scaled *COS_base_* as the baseline, and dynamically adjusts the highest-priority threshold according to the deviation between the measured highest-priority transmission success rate and the target value, through multiple stages including multiplicative halving, additive growth, and fine-tuning. The remaining priority thresholds are set proportionally based on the actual number of packets sent in the statistical period.

#### 5.1.3. Performance Metrics

This paper adopts the following four metrics to comprehensively evaluate protocol performance:

- Transmission success rate (Metric A): $\frac{\mathrm{Correctly received packets}}{\text{Upper-layer generated packets }}$, reflecting the end-to-end service completion rate.
- Transmission success rate (Metric B): $\frac{\mathrm{Correctly received packets}}{\mathrm{MAC-layer transmitted packets}}$, reflecting the collision probability of MAC-layer transmitted packets on the channel.
- End-to-end delay: The average time from when a packet is generated at the source node to when it is correctly received by the destination node.
- Throughput: The total amount of valid data successfully transmitted by the entire network per unit time.

The positioning of the two metrics, transmission success rate (Metric A) and transmission success rate (Metric B), needs to be clarified. Metric A reflects the end-to-end completion rate that upper-layer services truly care about; its loss includes both source-end active dropping and channel collisions, and it is the most objective and fair metric for evaluating protocol performance, and is therefore compared first in the simulation. Metric B reflects the transmission quality on the channel of packets that have obtained a transmission opportunity, and is used to separate the intermediate link of intra-channel collisions, serving as an auxiliary metric. It should be emphasized that if a protocol sends only a small number of packets to the channel, its collision probability is naturally low and Metric B will therefore be biased high, so Metric B should not be used as a standalone basis for judgment.

### 5.2. Simulation Results and Analysis

#### 5.2.1. Analysis of Important Parameters

This section further analyzes the short-window sensitivity and the long-window scaling-law properties of the proposed protocol. The simulation is based on the five-segment load scenario shown in Table 4, i.e., the operating condition adopts a bidirectional ramp load, and the traffic undergoes the complete gradual process of light load → load rising → full load → load falling → light load.

**Sensitivity Analysis of the Short Window *W_s_***

**Figure 10:**
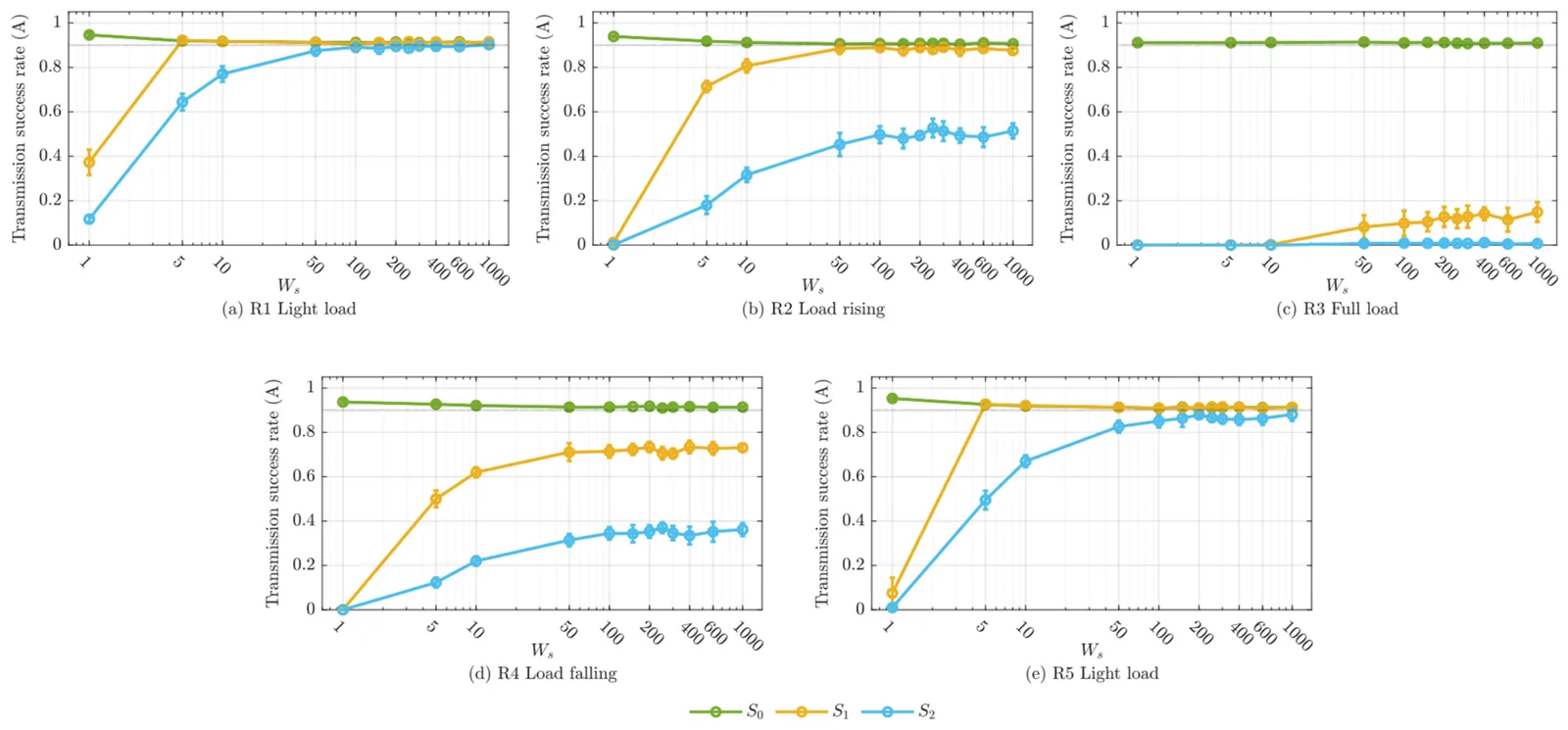
Transmission success rate (Metric A) of each priority across five load segments under different short-window lengths.

**Figure 11:**
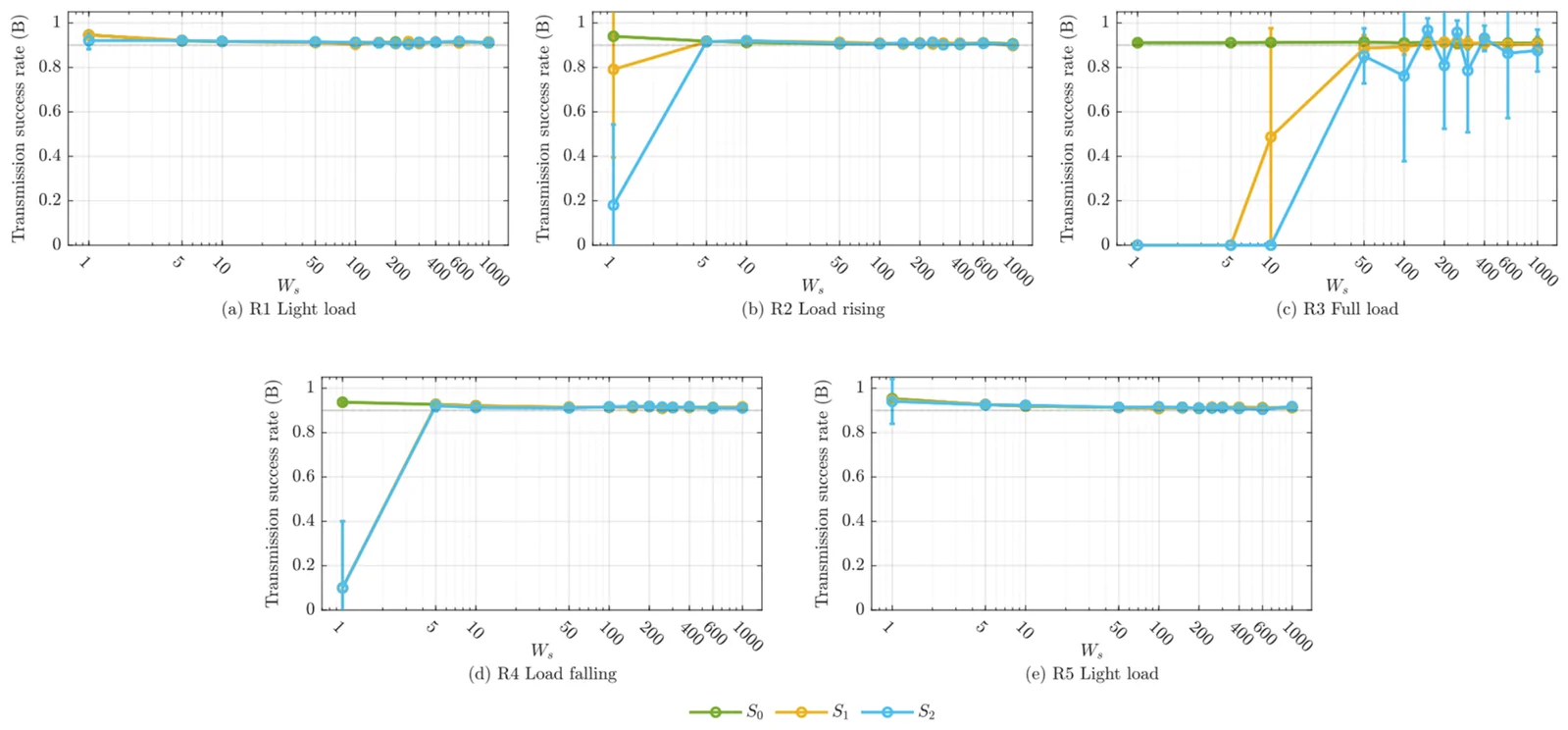
Transmission success rate (Metric B) of each priority across five load segments under different short-window lengths.

To verify the influence of the short window *W_s_* on protocol performance and to examine the short-window constraint given in Section 4.3.3, this group of simulations adopts the controlled-variable method, fixes the long window $W_{L}={W_{L}}^{0}=5000$ time slots, and performs a parameter sweep of the short window *W_s_* based on the five-segment ramp load scenario in Table 4. The sweep covers 12 values of *W_s_* ∈ {1, 5, 10, 50, 100, 152, 200, 250, 300, 400, 600, 1000} time slots, ranging from far below the statistical denoising lower bound (*W_s_* ≥ 48) to approximately 20% of the long-window design upper bound; 10 independent Monte Carlo simulations are performed for each parameter point. The transmission success rates under Metric A and Metric B for the three priorities in the five load segments are counted. The results are shown in Figure 10 and Figure 11, respectively.

From an overall perspective, the sensitivities of the three priorities to *W_s_* differ significantly. For the high priority *S*_0_, under all swept values, the Metric A and Metric B transmission success rates in the five load segments remain around 0.90, and the worst value does not change with *W_s_*, indicating that the short-window length has basically no influence on the high-priority metric. The low priorities *S*_1_ and *S*_2_ maintain relatively high transmission success rates in the light-load segments (R1, R5), decrease significantly as the load rises to the full-load segment (R3), and their performance strongly depends on whether *W_s_* crosses the statistical denoising lower bound; when *W_s_* is too small, they completely lose service capability.

Observing Metric A specifically (Figure 10), when *W_s_* ≤ 10 time slots, the Metric A of *S*_1_ and *S*_2_ in the full-load segment (R3) directly drops to 0, exhibiting the low-priority starvation phenomenon predicted by the theory in Section 4.3.3; when *W_s_* ≥ 50 time slots, the low-priority performance recovers. At *W_s_* = 200 time slots, the Metric A of *S*_1_ in the full-load segment (R3) reaches 0.130 ± 0.050, and the service capability is stable. In contrast, the worst Metric A of *S*_0_ across the five segments remains around 0.90 under all *W_s_*, indicating that the high-priority metric is insensitive to changes in the short-window length.

The results of Metric B (Figure 11) are consistent with the trend of Metric A: when *W_s_* ≤ 10 time slots, the Metric B of *S*_1_ and *S*_2_ in the full-load segment (R3) also drops to 0, indicating that starvation directly manifests as almost no packets being admitted for transmission at the source; after *W_s_* ≥ 50, normal operation is restored. It should be noted that the occasional high values of low-priority Metric B at individual *W_s_* values are merely because the few admitted packets happen to be successfully sent and the statistical denominator is extremely small, and do not reflect an improvement in delivery capability. The worst Metric B of *S*_0_ across the five segments also remains around 0.90, indicating that *S*_0_ packets entering the channel suffer almost no collision loss.

In the larger-value interval *W_s_* ∈ {250, 300, 400, 600, 1000} time slots, the worst Metric A of *S*_0_ across the five segments remains in the flat interval of 0.904~0.909, and further increasing *W_s_* brings almost no gain to high-priority performance; the Metric A of *S*_1_ and *S*_2_ in the full-load segment only increases slowly with *W_s_*. However, an excessively large *W_s_* weakens the instantaneous sensing capability of the short window and destroys the dual-window responsibility decoupling condition: when *W_s_* = 1000 time slots, the short-window length has reached 20% of the long-window upper bound $W_{L}^{0}$, and the responsibilities of the two-stage filtering overlap.

The above results mutually corroborate the hard lower bound of *W_s_* ≥ 48 time slots for statistical denoising given in Section 4.3.3. Taking into account the performance optimization constraint of dual-window responsibility decoupling, this paper selects *W_s_* = 200 time slots. This value not only satisfies the hard constraint of statistical denoising, but also satisfies *W_s_* ≪ *W_L_*, realizing short–long-window responsibility separation, stabilizing low-priority service performance, and avoiding unnecessary tracking lag.

**Scaling Law Analysis of the Long Window *W_L_***

**Figure 12:**
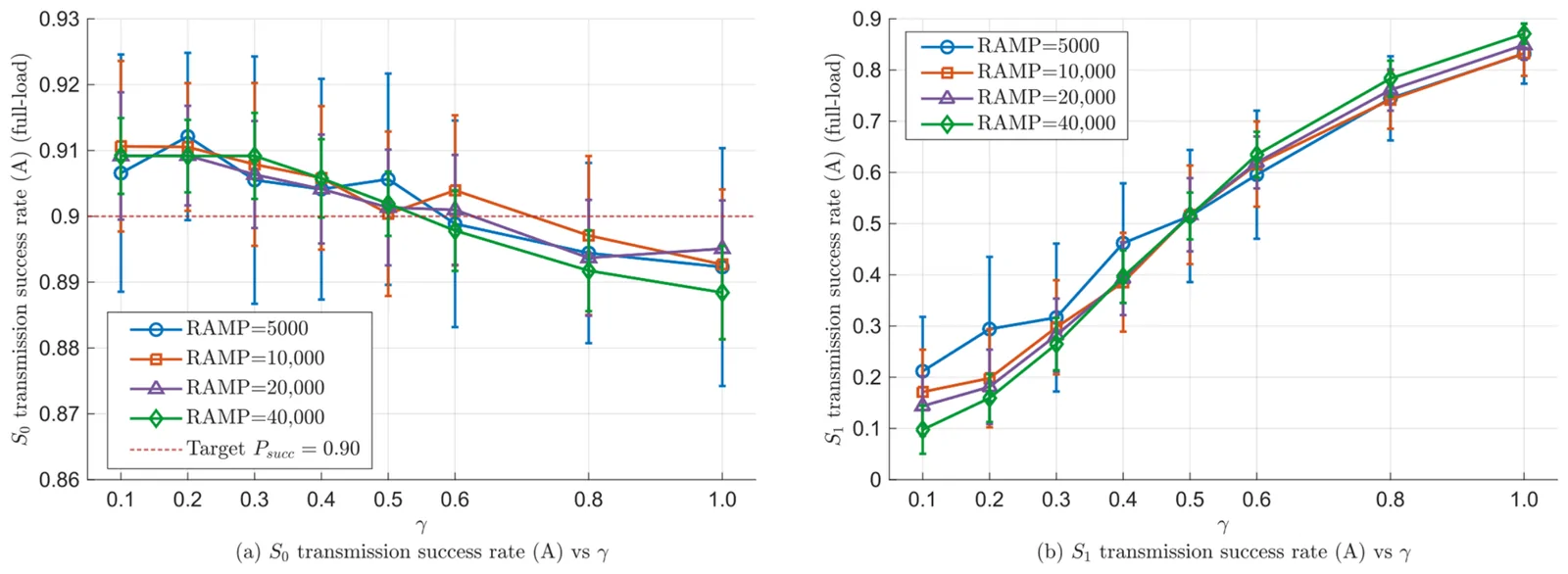
Long-window lag effect: transmission success rate (Metric A) of *S*_0_ and *S*_1_ as a function of *γ*.

To verify the scaling law corresponding to the long-window upper bound constraint *W_L_* ≤ *γ* ⋅ *RAMP*, this group of simulations fixes *W_S_* = 200 time slots and adjusts the load change slope by changing the ramp duration *RAMP*; the value set of *RAMP* is {5000, 10,000, 20,000, 40,000} time slots, and a smaller *RAMP* represents a more drastic load change. For each *RAMP* value, according to *γ* = *W_L_*/*RAMP*, *γ* ∈ {0.1, 0.2, 0.3, 0.4, 0.5, 0.6, 0.8, 1.0} is traversed, thereby obtaining 32 long-window *W_L_* values; at each *W_L_*, 40 independent simulations with different random seeds are performed, and then the transmission success rates (Metric A) of the two services *S*_0_ and *S*_1_ in the full-load R3 segment are counted. The results are shown in Figure 12.

Figure 12 reveals the influence pattern of *γ* on system performance. A key experimental phenomenon is that under different load variation time scales *RAMP*, the inflection points at which the *S*_0_ transmission success rate drops significantly are concentrated near the same *γ*. This phenomenon directly corroborates the core inference of the scaling law: the system performance constraint is dominated by the dimensionless proportional coefficient *γ*, rather than by the absolute long-window length *W_L_*.

From the *S*_0_ performance curve, the Metric A of *S*_0_ in the full-load segment remains overall near the 90% target success rate when *γ* ≤ 0.5; when *γ* increases to 1.0, the *S*_0_ success rate under all operating conditions falls below the 90% target.

When *γ* is too small, the corresponding *W_L_* is relatively small, the EWMA statistical noise level increases, the admission probability undergoes high-frequency oscillation, low-priority services are excessively throttled, and the *S*_1_ transmission success rate is relatively low; when *γ* is too large, the corresponding *W_L_* is relatively large, the ramp tracking deviation of the filter increases, the true channel occupancy is underestimated during the load rising stage, low priorities are excessively admitted, encroaching on the channel resources of high-priority services, causing *S*_0_ performance loss.

Although *S*_0_ can still meet the metric when *γ* ≤ 0.5, considering that during the subsequent operation of the adaptive long window, *γ* has a risk of instantaneous crossing due to online estimation error, a conservative upper bound needs to be selected. Comprehensively weighing high-priority reliability and low-priority service capability, this paper takes the conservative tracking lag upper bound *γ_max_* = 0.2, i.e., *W_L_* ≤ 0.2·*RAMP*. At this time, the Metric A of *S*_1_ in the full-load R3 segment is in the interval of 0.16~0.29, and low priorities can obtain moderate service capability.

It should be noted that the *S*_0_ and *S*_1_ performance in the full-load R3 segment counted in this subsection reflect the transient convergence performance after the R2–R3 load ramp transition. A larger *γ* represents a longer long-window memory duration, and the EWMA filter converges more slowly after the load ramp switching; the performance loss caused by transient tracking deviation is more obvious. This simulation phenomenon is homologous to the tracking deviation mechanism analysis in Section 4.3.4, supporting the validity of the scaling law $W_{L}^{*}=\gamma \cdot \mathrm{RAMP}$.

**Robustness Analysis of the Calibration Constant *K* and Smoothing Coefficients**

The calibration constant *K* is used to convert the severity of the load variation output by the dual-EWMA difference into the time scale of the load variation, and the time constants of the dual-EWMA smoothing coefficients determine the response speed of the two filters; together they determine the adjustment behavior of the long-window length. To examine the robustness of their values, this paper conducts separate comparative experiments on the two types of parameters under the same five-segment load scenario, with 10 independent simulations in each group. The two experiments share the same baseline configuration, that is, *K* = 50,000; the fast time constant *τ_f_* is 200 time slots and the slow time constant *τ_s_* is 5000 time slots. For *K*, the baseline value and half the baseline value are compared. As shown in Table 7, the maximum difference in the five-segment transmission success rate (Metric A) is 0.0038, occurring in segment R5, which is smaller than the within-segment standard deviation of repeated simulations in the same scenario, 0.0042–0.0098. For the dual-EWMA smoothing coefficients, the fast coefficient is set to 100, 200, and 400 time slots (with the slow one fixed at 5000 time slots) and the slow coefficient to 2500, 5000, and 10,000 time slots (with the fast one fixed at 200 time slots), giving five configurations in total, as shown in Table 8. Under the four deviating configurations, the maximum difference in the end-to-end completion rate of the highest priority across the five load segments relative to the baseline configuration is 0.0041, occurring in segment R1 when the fast coefficient is 100 time slots, which is smaller than the within-segment standard deviation of repeated simulations in the same scenario, 0.0065–0.0074; the completion rates of the second-highest and lowest priorities in the full-load segment are also close to each other across the configurations. The results for the two types of parameters consistently show that the algorithm performance is insensitive to the values of the calibration constant and the smoothing coefficients. The reason is that *K* only affects the estimation scale of the load variation time scale and the two smoothing coefficients only determine the adjustment speed of the long-window length; neither directly participates in the admission decision, so a change in their values causes only a slight difference in the load tracking speed.

#### 5.2.2. Algorithm Comparison Analysis: Eight-Node Single-Channel

The simulations in this section are based on the six-segment load scenario shown in Table 5, comparing the transmission success rates (Metrics A and B), end-to-end delay, and throughput of four algorithms under R1 light load → R2 load rising → R3 full load → R4 load rising → R5 heavy load → R6 load falling. For the DW-PA-SPMA protocol proposed in this paper, the short window takes a fixed value *W_s_* = 200 time slots; the long window enables the dual-EWMA differential adaptive mechanism in Section 4.3.5, and *W_L_* is dynamically determined by the online estimation result of the load variation time scale combined with the calibration coefficient *γ_max_* = 0.2, with the long-window upper bound set to $W_{L}^{0}$ = 5000 time slots. All four algorithms are simulated with 10 independent simulations using different random seeds.

**Transmission Success Rate (Metric A)**

**Figure 13:**
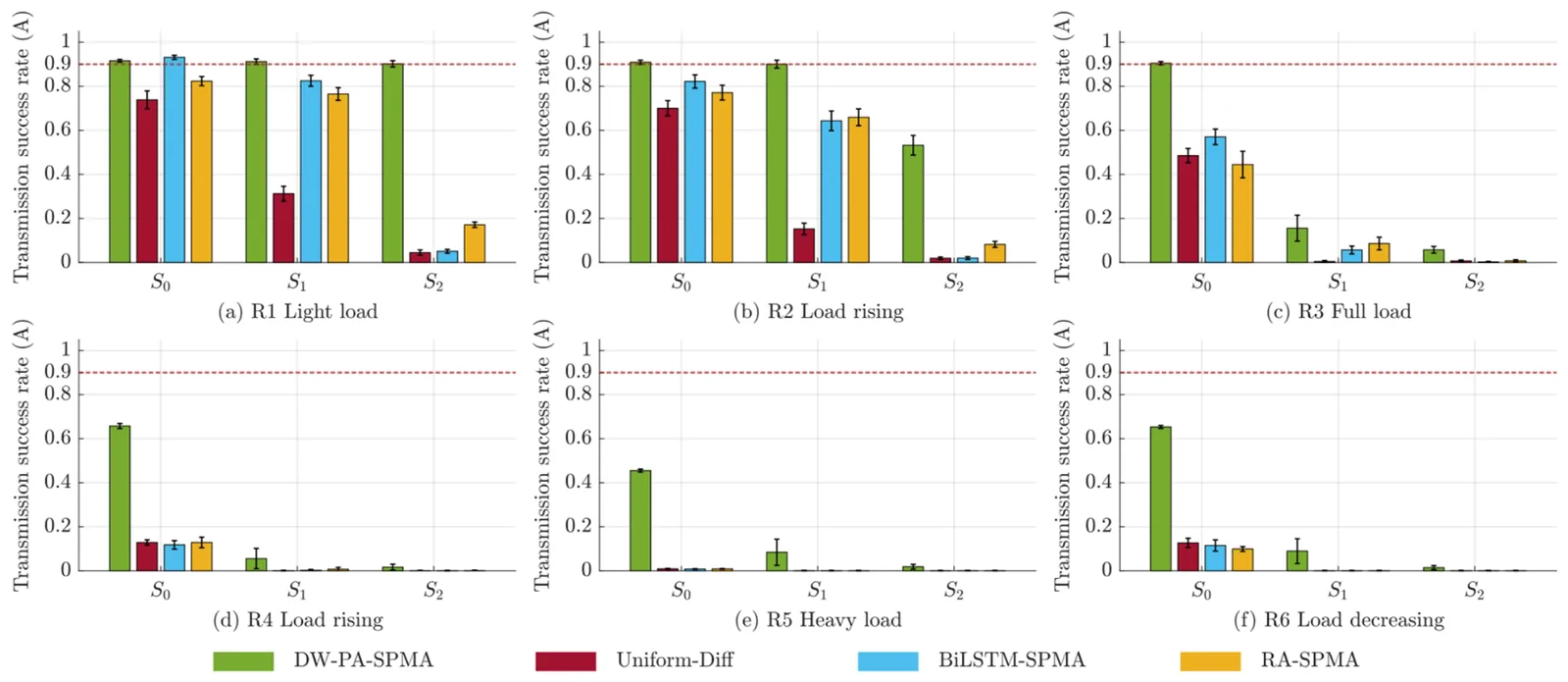
Comparison of transmission success rate (Metric A) across different load segments.

Figure 13 presents the Metric A transmission success rates of the four algorithms in the six load segments. Metric A reflects the end-to-end service completion rate of packets generated by the upper-layer service, and its value is jointly affected by two parts: source-end admission dropping and channel collision failure.

**Figure 14:**
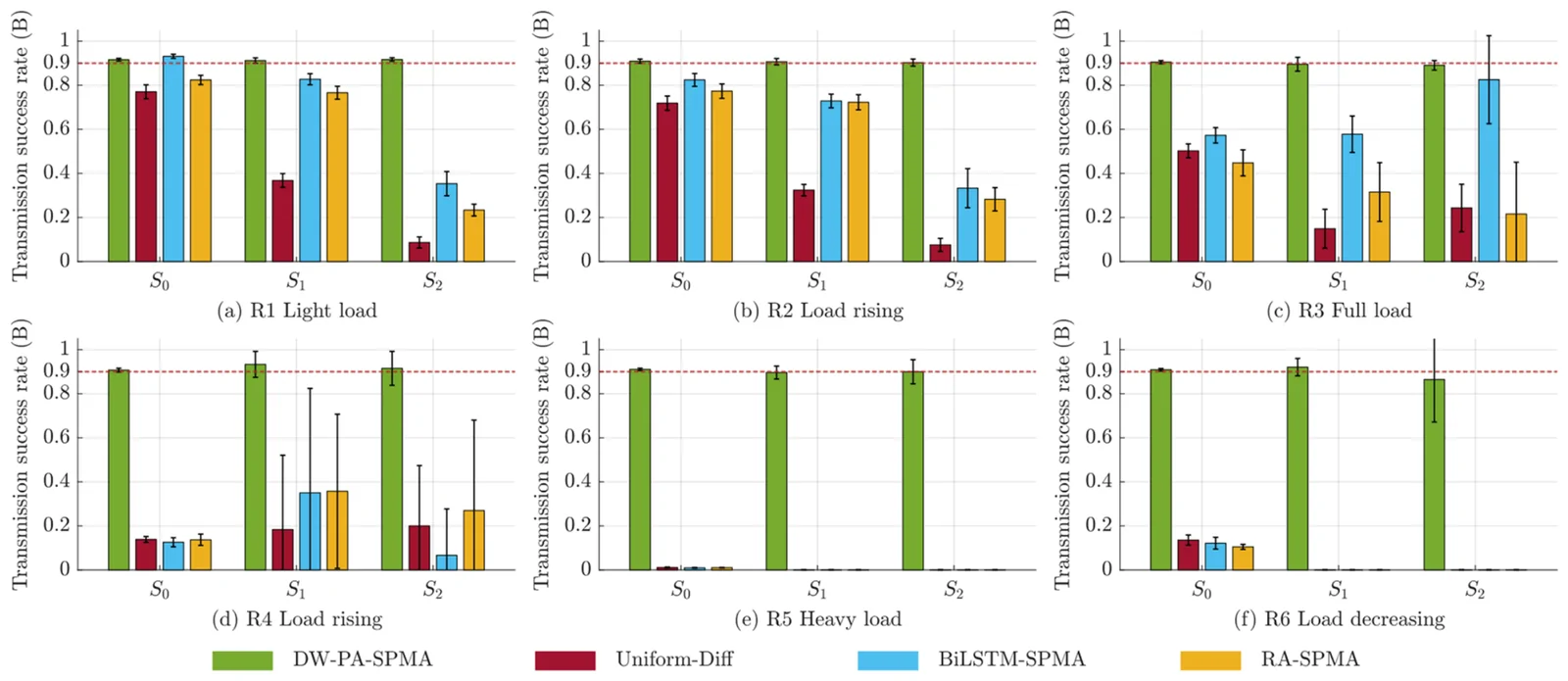
Comparison of transmission success rate (Metric B) across different load segments.

For DW-PA-SPMA, the Metric A of *S*_0_ in the light-load to full-load segments (R1–R3) stably remains at 90.5~91.6%, satisfying the preset target; after entering the heavy-load R5 segment, it drops to about 0.45. This decrease mainly comes from the flow-limiting effect of the source-end probabilistic admission mechanism: in the R5 segment, the *S*_0_ service arrival volume reaches 2.0 times the full load, and the steady-state admission probability calculated by the capacity budget has a mean value of about 0.5. A large number of excess packets are directly discarded at the packet generation source and no longer enter the MAC queue or the channel; packets that enter the channel after admission screening can still maintain good transmission quality, corresponding to the *S*_0_ Metric B remaining at about 0.91 (see Figure 14). Therefore, the decrease in Metric A in the heavy-load segment is an expected trade-off brought by source-end traffic shaping, not a channel-level transmission failure. In contrast, the three comparison algorithms lack source-end proactive flow-limiting capability. Under the light-load R1 condition, the performance of BiLSTM-SPMA is slightly better than that of the proposed algorithm, while RA-SPMA and Uniform-Diff have already shown performance degradation; from the full-load R3 segment onward, the *S*_0_ Metric A of the three comparison algorithms all decline significantly; in the heavy-load R5 segment, the highest-priority transmission success rates are all less than 1%, proving that the traditional threshold–backoff-based SPMA mechanism basically fails under overload scenarios; in the load-falling R6 segment, performance only partially recovers.

For the low-priority services *S*_1_ and *S*_2_, the proposed algorithm can guarantee a certain delivery capability in the light-load segment; in the full-load R3 segment, the Metric A of *S*_1_ and *S*_2_ is about 0.16 and 0.06, respectively; after entering R4 and subsequent segments, the low-priority Metric A drops to a low level of 0.02–0.09. The relatively low low-priority performance is an inevitable result of the “highest priority first” design: when service demand exceeds the remaining channel capacity, excess packets are probabilistically discarded at the source, and channel resources are preferentially tilted toward *S*_0_. The low-priority Metric A of the three comparison algorithms all drop to 0 in the heavy-load segment.

Metric A simultaneously includes two types of loss: source-end dropping and channel collision. To separate the true intra-channel transmission quality, the Metric B transmission success rate is further observed, as detailed in the next subsection.

**Transmission Success Rate (Metric B)**

Metric B characterizes the reception success rate of packets that have already obtained transmission eligibility at the MAC layer and are actually sent to the channel. This metric eliminates the influence of source-end admission dropping and purely reflects the transmission quality of intra-channel packets subject to collision interference. The simulation results are shown in Figure 14.

For the DW-PA-SPMA protocol, the Metric B of *S*_0_ stably remains at 0.90~0.92 in all six load segments. In the light-load to full-load segments (R1–R3), the values of Metric A and Metric B almost coincide, indicating that there is very little source-end admission dropping at this stage, and packet loss almost entirely comes from channel collisions; in the heavy-load R5 segment, although Metric A drops to about 0.45, Metric B still remains at about 0.91. This phenomenon directly verifies the core effect of source-end probabilistic admission shaping: even if the upper-layer service is 2× overloaded, after source-end probabilistic admission screening, the total amount of packets actually entering the channel is constrained within the channel capacity budget. The intra-channel collision level does not deteriorate with the external arrival traffic, and the channel transmission quality of high-priority packets is stably guaranteed. In contrast, the three comparison algorithms, lacking a source-end proactive traffic shaping mechanism, accumulate packets and then send them in concentrated bursts, causing sharp deterioration of channel collisions. In the light-load R1 segment, the Metric B of BiLSTM-SPMA is still acceptable, while RA-SPMA and Uniform-Diff are already below the 90% target; after entering the full-load R3 segment, the Metric B of all comparison algorithms declines significantly; in the heavy-load R5 segment, it plummets to about 0.01, and the protocols almost completely fail; in the load-falling R6 segment, performance only partially recovers.

The above simulation results indicate that the traditional SPMA post-hoc threshold–backoff architecture easily forms a vicious cycle of packet accumulation-concentrated transmission-aggravated collision, whereas DW-PA-SPMA effectively avoids this effect by relying on source-end probabilistic admission shaping.

Metric A and Metric B evaluate the end-to-end delivery capability and the intra-channel transmission quality, respectively. Next, the end-to-end delay characteristics of each algorithm are further analyzed.

**End-to-End Delay**

**Figure 15:**
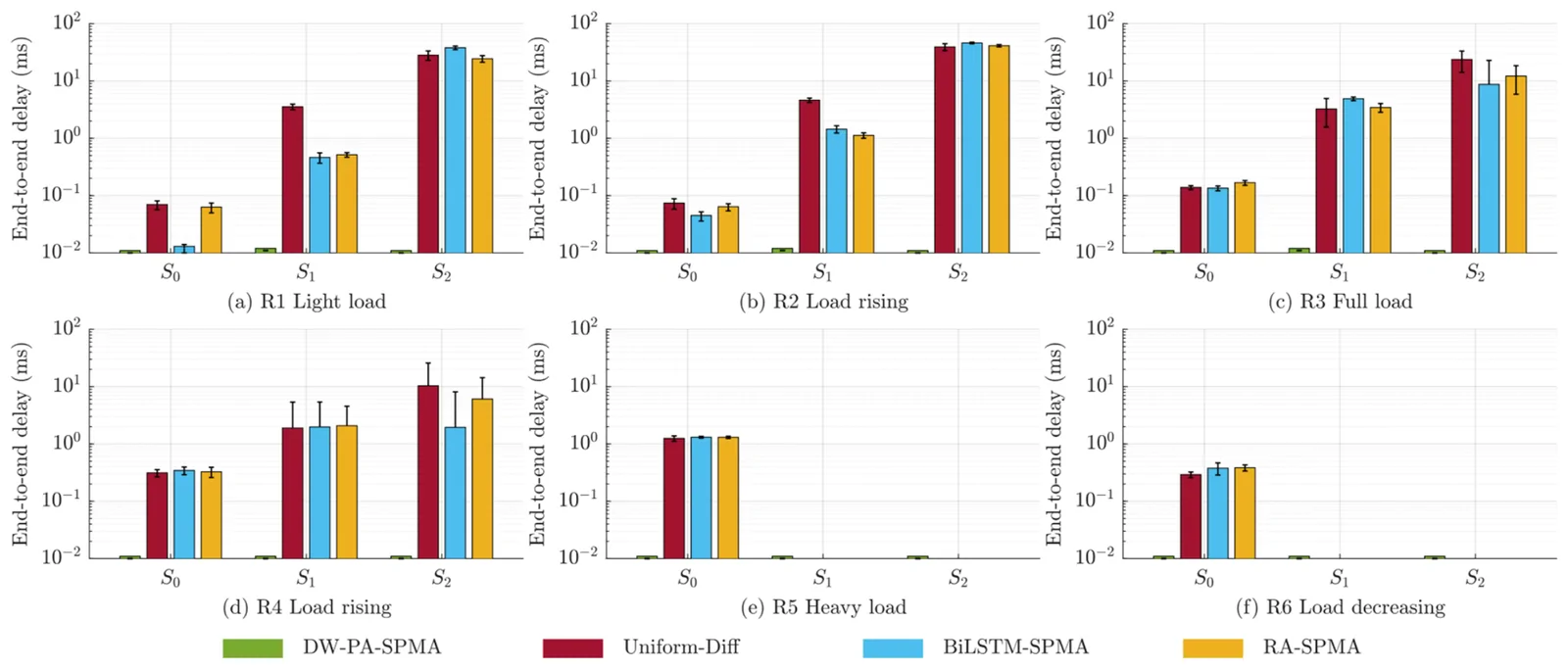
Comparison of end-to-end delay across different load segments.

End-to-end delay is defined as the time experienced from the moment a packet is generated at the source node service layer to the moment it is correctly received by the destination node, including all time consumption such as queuing, backoff, and channel transmission. The statistics are collected only for packets that are finally correctly received by the destination node; failed packets are not counted in the delay average, which is consistent with the conventions of the two success-rate metrics and avoids ambiguities such as treating the delay of failed packets as infinite. The delay statistics are aggregated separately for each load segment and each priority, in milliseconds, with a time-slot length of 10 μs. The simulation is a time-slot-driven discrete-event model: each packet records its generation time slot *t*_gen_ at generation, and the destination node records the reception time slot *t*_recv_ upon correct reception; the delay of the packet is (*t*_recv_ − *t*_gen_) × *T*_slot_, where *T*_slot_ = 10 μs is the time-slot length. Since the scheduling mechanism of this paper is that each node makes the decision and transmits at the beginning of each time slot, and successful reception is completed at the destination within the same or the immediately adjacent time slot, the discrete values of the delay are integer multiples of a time slot, with a minimum of one time slot. The event order within a time slot (generation, admission decision, enqueuing, scheduling transmission, reception) is fixed to ensure the reproducibility of the timing convention. The simulation results are shown in Figure 15.

For DW-PA-SPMA, under all load segments and all priority services, the end-to-end delay stably remains at about 0.01 ms (1 time slot). This result verifies the effect of the “admission means transmission” scheduling mechanism: after source-end probabilistic admission, packets directly enter the queue, and there is no multi-level backoff retry; at the same time, the source-end flow-limiting mechanism maintains the number of queued packets at a very low level, so queuing accumulation hardly occurs. After a packet is enqueued, it can be scheduled for transmission in the next time slot. Therefore, the end-to-end delay is constrained to the theoretical lower limit of a single time slot, and the delay characteristic is basically decoupled from the traffic load level.

The three comparison algorithms are affected by queue accumulation and random backoff mechanisms, and their delays increase sharply as the traffic load rises. Taking the full-load R3 segment as an example, the end-to-end delay of the *S*_0_ service reaches 0.13~0.17 ms, and the delay of the *S*_1_ service further increases to 3~5 ms; after entering the heavy-load R5 segment, the *S*_0_ delay rises to 1.25~1.31 ms, at which point *S*_1_ and *S*_2_ have almost no successfully received packets. Traditional SPMA places control post-hoc at the transmission time slot; under overload conditions, a large number of packets accumulate in the queue and repeatedly retry backoff, directly causing significant delay expansion.

In addition to transmission success rate and delay, the effective throughput of the entire network is also an important metric for measuring the resource utilization efficiency of MAC protocols. The throughput performance of each algorithm is analyzed below.

**Throughput**

**Figure 16:**
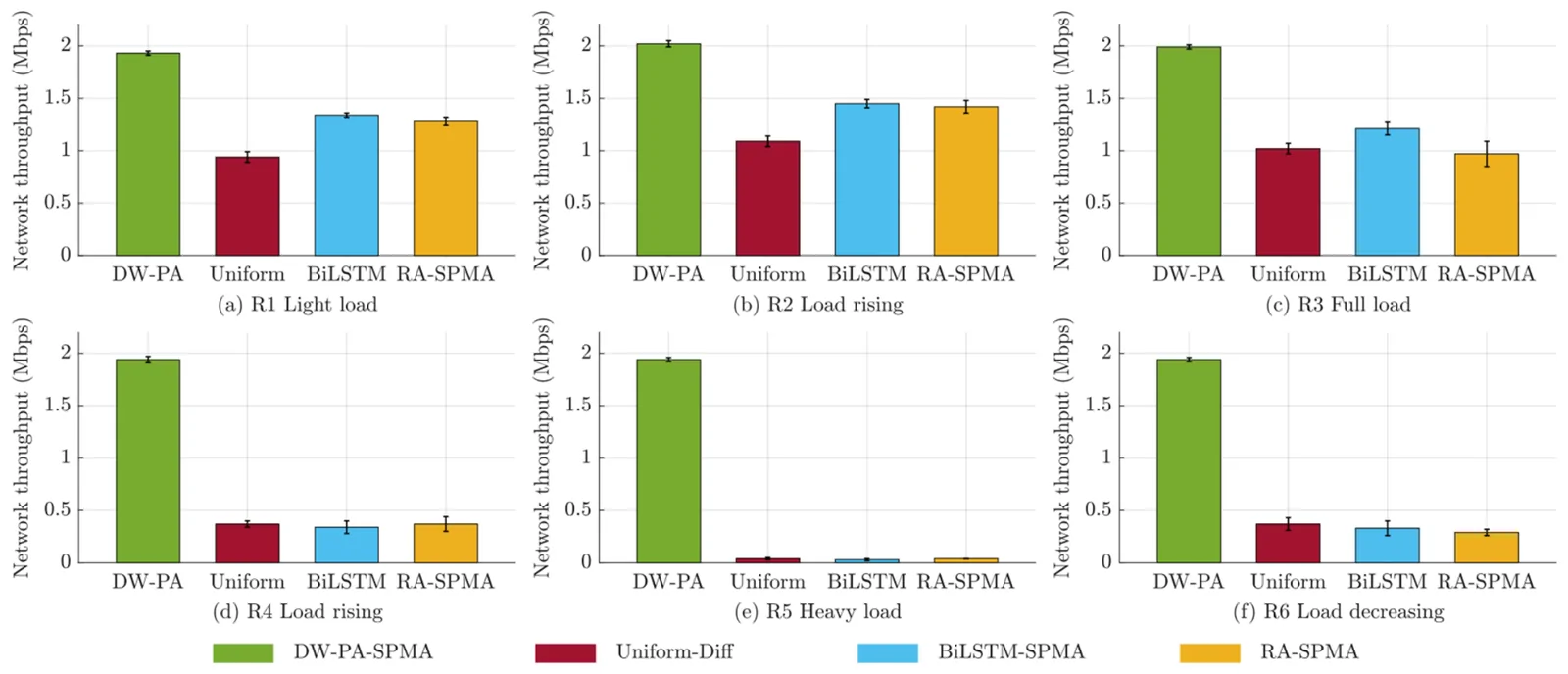
Comparison of throughput across different load segments.

In this paper, throughput refers to the effective service data volume successfully received by all nodes in the entire network per unit time, reflecting the actual utilization efficiency of channel resources. The simulation results are shown in Figure 16.

The throughput of DW-PA-SPMA rises from about 1.93 Mbps in the light-load segment (R1) to a peak of about 2.02 Mbps in the load-rising segment (R2), and thereafter stabilizes at 1.94–1.99 Mbps from the full-load to the load-falling segments (R3–R6), no longer continuing to grow with the upper-layer service arrival volume. This phenomenon conforms to the protocol design expectation: when the service exceeds the channel carrying upper limit, excess packets are discarded at the source, and the total traffic entering the channel is constrained within the channel capacity budget. Therefore, the throughput reaches a saturation plateau and no longer rises with external service input.

In contrast, the throughput of the three comparison algorithms is lower than that of the proposed algorithm in every load segment. In the light-load segment (R1), RA-SPMA, BiLSTM-SPMA, and Uniform-Diff achieve 0.94, 1.34, and 1.28 Mbps, respectively, only 49%, 69%, and 66% of the proposed algorithm (1.93 Mbps); in the load-rising segment (R2), they achieve 1.09, 1.45, and 1.42 Mbps, i.e., 54%, 72%, and 70% of the proposed algorithm; in the full-load segment (R3), they achieve 1.02, 1.21, and 0.97 Mbps, i.e., 51%, 61%, and 49% of the proposed algorithm (1.99 Mbps). After entering the R4–R6 segments, the throughput of the three comparison algorithms collapses successively: in the heavy-load segment (R5), it falls to 0.03–0.04 Mbps, only about 2% of the proposed algorithm, and in the load-rising and load-falling segments (R4, R6), it is only 15–19% of the proposed algorithm. Traditional SPMA lacks source-end traffic shaping; under overload scenarios, packets accumulate massively and burst in a concentrated manner, the channel collision probability rises sharply, a large number of packets fail in transmission, causing channel resources to be occupied by invalid packets and effective throughput to collapse substantially.

The aforementioned metrics evaluate protocol performance from the service level. To further reveal the underlying statistical mechanism brought by source-end probabilistic admission shaping, the interval distribution of packets actually sent on the channel is statistically tested below.

**Statistical Characteristics of Channel Data**

**Figure 17:**
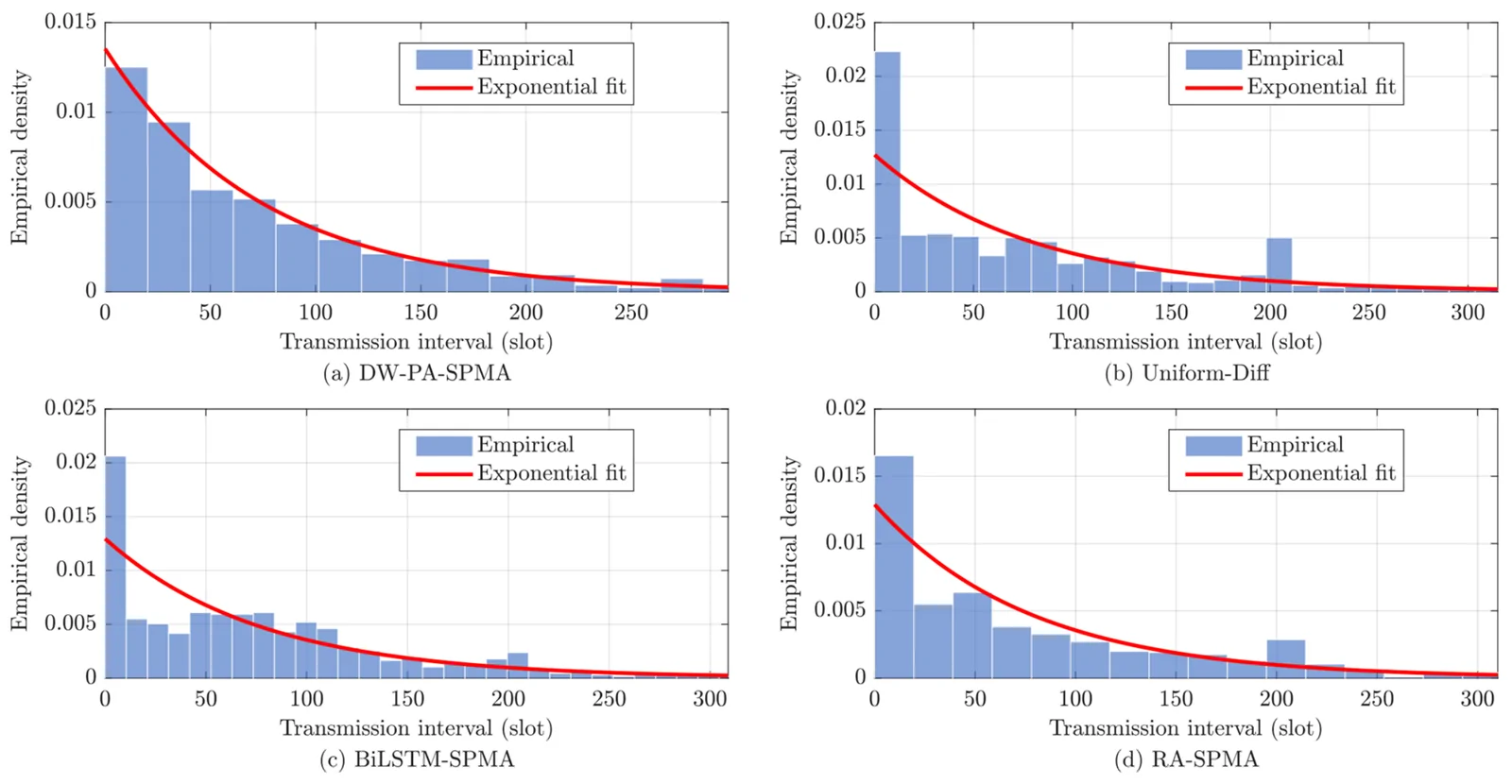
Coefficient of variation of the *S*_0_ data sent to the channel by node 1 in the full-load segment.

**Figure 18:**
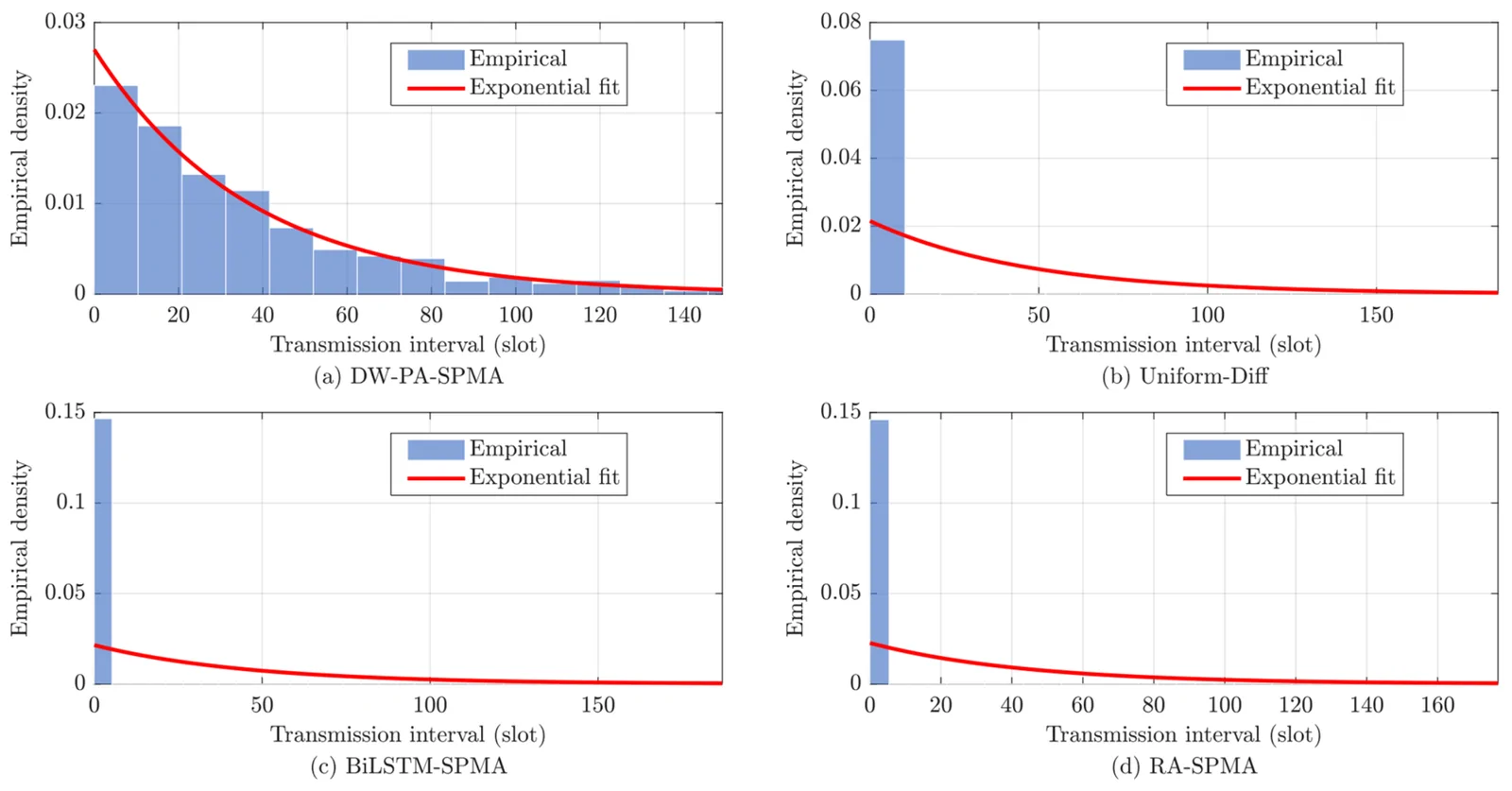
Coefficient of variation of the *S*_0_ data sent to the channel by node 1 in the heavy-load segment.

The statistical characteristics of packet transmission intervals are the basic premise of the analytical modeling of SPMA-type protocols. The classical SPMA theoretical derivation is built on the assumption that the packets sent on the channel follow a Poisson process. Taking node 1 as a representative, this section collects the packet intervals of the highest-priority service actually sent by this node to the channel, and further examines whether it maintains Poisson statistical characteristics from four aspects: the coefficient of variation, the Kolmogorov–Smirnov (KS) goodness of fit, the skewness and kurtosis, and the autocorrelation and Ljung–Box independence. The coefficient of variation results are shown in Figure 17 and Figure 18.

For the proposed algorithm, taking node 1 as a representative, 677 packet interval samples are collected in the full-load segment, with a mean interval of 73.84 time slots and a coefficient of variation of 1.051; in the heavy-load segment there are 1076 samples, with a mean interval of 37.02 time slots and a coefficient of variation of 1.050. The coefficients of variation in the two statistics are both close to the expected value of 1 for the exponential distribution. This result is consistent with the mechanism: in the full-load segment the mean packet arrival interval of each node is 76 time slots, and after shaping by source-end probabilistic admission according to the capacity budget, the interval of the packets sent by the node is correspondingly relaxed; in the heavy-load segment the traffic arrivals double relative to the full-load segment and the admission probability is tightened accordingly, yet the mean interval of the packets sent on the channel still remains stable at the level of tens of time slots, consistent with the theoretical capacity constraint. Further statistical tests support this judgment: the Kolmogorov–Smirnov statistic with the exponential distribution as the theoretical baseline is 0.0356 and 0.0306 in the full-load and heavy-load segments, respectively, both smaller than the critical values of 0.0523 and 0.0415 at the 0.05 significance level, so the test cannot reject the exponential distribution hypothesis; the absolute values of the lag-1 to lag-3 autocorrelation coefficients of the interval sequence do not exceed 0.05, and the *p*-values of the Ljung–Box test are 0.511 and 0.386, respectively, so the null hypothesis that the intervals are mutually independent cannot be rejected. The *p*-value is the probability of obtaining the current or a more extreme test result when the null hypothesis holds, and its exact value is obtained here by a parametric bootstrap method (generating equal-length exponential interval sequences according to the parameters estimated from the samples and reconstructing the empirical distribution of the KS statistic). The above results show that after shaping by source-end probabilistic admission, the interval distribution of the packets sent by the node is consistent with a Poisson process.

In contrast, for the three comparison algorithms, affected by the post-hoc threshold decision and random backoff mechanisms, packets are prone to accumulation and concentrated bursts, and the transmission intervals deviate significantly from the Poisson distribution: in the full-load segment the coefficients of variation of the packet intervals of Uniform-Diff, BiLSTM-SPMA, and RA-SPMA are 1.085, 0.945, and 1.112, respectively, rising to 1.842, 1.828, and 1.889 in the heavy-load segment; the Kolmogorov–Smirnov statistics are 0.2047, 0.1507, and 0.1980 in the full-load segment and further rise to 0.7492, 0.7465, and 0.7619 in the heavy-load segment, all far larger than the critical value of about 0.05 at the 0.05 significance level, so the test significantly rejects the exponential distribution hypothesis. The proportions of intervals equal to 1 time slot are 21.73%, 16.36%, and 21.09% (full-load segment) and 77.05%, 76.78%, and 78.43% (heavy-load segment), that is, a large number of packets are transmitted continuously in adjacent time slots, whereas this proportion is only 2.22% and 2.88% for the proposed algorithm; the lag-1 to lag-3 autocorrelation coefficients of the interval sequences of the three comparison algorithms in the heavy-load segment reach −0.25 to −0.07, and the *p*-values of the Ljung–Box test are less than 10^−3^, showing significant negative correlation. These features indicate that their transmission process has clearly deviated from a Poisson process, which is a direct manifestation of the concentrated bursts after packet accumulation.

**Robustness to Baseline Threshold Calibration Deviation**

**Figure 19:**
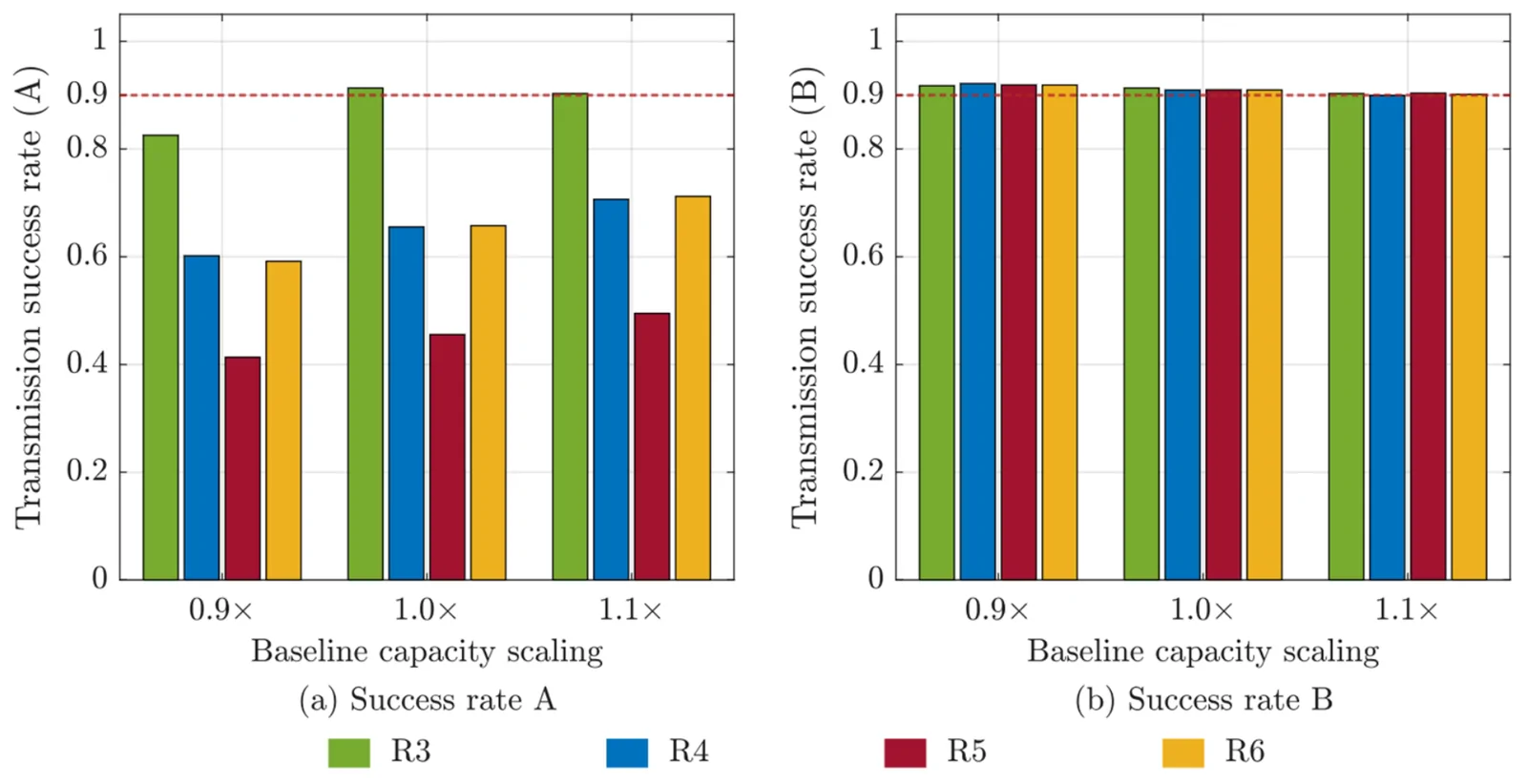
Performance comparison under the baseline threshold calibration deviation.

To verify the sensitivity of the algorithm to the baseline threshold calibration deviation, this paper conducts 10 independent simulations under the six-segment load scenario for three configurations of the baseline threshold, namely 0.9×, 1.0×, and 1.1× the measured calibration value. The baseline threshold in this paper can be obtained either by theoretical calculation using Formula (2) or by measurement-based calibration. The measured value 21.64 is about 2.7% higher than the theoretical value 21.07, and the deviation originates from the quantization granularity with a step of four time slots and the Poisson fluctuation within a finite statistical window. The simulation results are shown in Figure 19. Figure 19a,b respectively give the end-to-end transmission success rate (Metric A) and the intra-channel transmission quality (Metric B) of the highest priority under the three baseline threshold ratios, and for each ratio a comparison of the four load segments R3, R4, R5, and R6 is given.

When the baseline threshold is 10% smaller, the Metric A of the highest priority decreases markedly in all high-load segments: in the full-load segment R3 it drops from 0.913 to 0.825, in R4 from 0.655 to 0.602, in the heavy-load segment R5 from 0.456 to 0.414, and in R6 from 0.658 to 0.592; meanwhile the intra-channel transmission quality (Metric B) generally increases, with R3 rising from 0.913 to 0.918, R4 from 0.909 to 0.922, and R5 from 0.910 to 0.919. This indicates that a smaller baseline threshold over-tightens the admission probability and increases source-end dropping, reducing the end-to-end completion rate while improving the intra-channel collision level. When the baseline threshold is 10% larger, the trend is opposite: the Metric A in segments R4 to R6 generally rises to 0.495–0.712, while in the full-load segment R3 it decreases slightly from 0.913 to 0.903, and the Metric B generally decreases slightly, with R4 dropping from 0.909 to 0.900 and R5 from 0.910 to 0.904. When the calibration is accurate, the Metric A in the full-load segment is 0.913, higher than on both sides, indicating that the calibration accuracy of the baseline threshold directly determines the guarantee level of the highest priority. The above results show that the effect of the calibration deviation is a trade-off between the end-to-end completion rate and the intra-channel transmission quality: an underestimated calibration significantly weakens the end-to-end completion rate of the highest priority in all high-load segments, while an overestimated calibration slightly increases intra-channel collisions in the heavy-load segment and slightly reduces the completion rate in the full-load segment. Therefore, the calibration accuracy of the baseline threshold must be ensured in actual deployment. The calibration deviation in this paper is about 2.7%, only about one quarter of the perturbation magnitude, and if necessary the calibrated value can be slowly self-corrected according to the long-term observed deviation of the highest-priority success rate.

**Robustness to Non-Poisson Inputs**

To examine the behavior of the proposed mechanism when the service arrival process deviates from the Poisson assumption, this section changes the distribution of the service arrival interval from a negative exponential distribution to a Pareto heavy-tailed distribution (shape parameters α = 1.5 and 2.5, with the same mean as the Poisson input) and to periodic arrivals (same mean interval, jitter coefficient 0.2), and repeats the experiments under the six-segment load scenario, with 10 independent simulations for each configuration.

**Figure 20:**
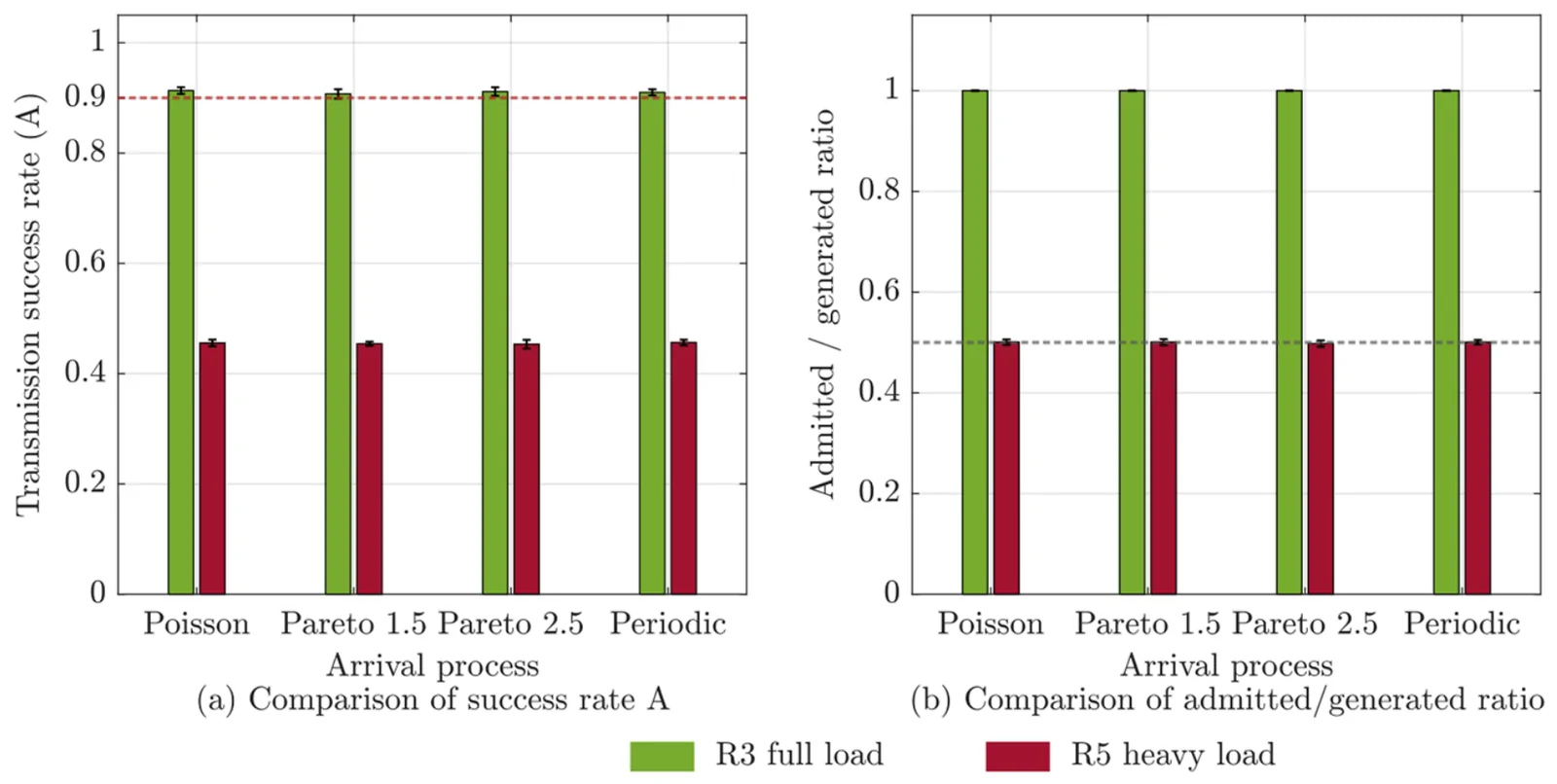
Performance comparison of the highest priority under the four arrival processes.

Figure 20a compares the transmission success rate (Metric A) of the highest priority in the full-load segment R3 and the heavy-load segment R5 under the four different arrival processes; Figure 20b gives the ratio of the admitted traffic to the generated traffic of the highest priority in the full-load segment R3 and the heavy-load segment R5 under the four different arrival processes.

As shown in the figure, under the three non-Poisson inputs the transmission success rate (Metric A) in the heavy-load segment R5 is 0.454 (Pareto α = 1.5), 0.453 (Pareto α = 2.5), and 0.457 (periodic arrivals), respectively, essentially the same as 0.456 under the Poisson input; the range of the four values is only 0.003 (0.4534–0.4565), smaller than the minimum of the within-segment standard deviations of the four groups: 0.0036. More importantly, the ratio of the admitted traffic to the generated traffic under the three non-Poisson inputs remains around 0.50 (0.498–0.501), the same as 0.501 under the Poisson input, showing that source-end probabilistic admission still constrains the traffic injected into the channel within the capacity budget; this indicates that the computational structure of the capacity budget depends only on the mean arrivals of each priority and not on the form of the arrival distribution. In the full-load segment R3, the transmission success rate (Metric A) of the highest priority under the four arrival processes is 0.913 (Poisson), 0.907 (Pareto α = 1.5), 0.911 (Pareto α = 2.5), and 0.910 (periodic), respectively, with a range of 0.006, which is of the same order as the within-segment standard deviations of the four arrival processes (0.0056–0.0085); the ratio of the admitted traffic to the generated traffic is 1.000 for all four arrival processes, because the total traffic arrivals in the full-load segment do not yet exceed the capacity budget and source-end admission drops no packets; after entering the heavy-load segment R5, the ratio uniformly drops to about 0.50, indicating that the part exceeding the capacity budget is removed at the source in the same proportion. It can be seen that from full load to heavy load, the shaping ratio of admission control on traffic is entirely determined by the ratio of the mean traffic arrivals to the capacity budget, irrespective of the distribution followed by the arrival interval.

The above only gives the comparison of the transmission success rate (Metric A) of the highest priority under different arrival processes. In fact, the comparison results of the transmission success rate (Metric B) and the end-to-end delay of the highest priority, the transmission success rates (Metrics A and B) and the end-to-end delays of the other priorities, and the throughput over the six load segments likewise show that the proposed mechanism does not depend on the form of the arrival distribution. For example, the Metric B of the highest priority under the four arrival processes and six load segments lies between 0.8999 and 0.9191; compared with 0.9095–0.9133 under the Poisson input, the maximum deviation is 0.0064 (Pareto α = 2.5 in segment R1), which is of the same order as the within-segment standard deviations of the four arrival processes (0.0045–0.0105). The end-to-end delay of the highest priority under the four arrival processes and six load segments is constantly 0.0100 ms (that is, one time slot), indicating that the zero-queuing property brought by “admission means transmission” is independent of the arrival distribution; the end-to-end delays of the second-highest and lowest priorities also remain at the level of 10 μs (0.006–0.011 ms). It should be noted that the second-highest and lowest priorities are actively suppressed to very low success rates in the heavy-load segment, which is precisely the result of the priority-layered reservation of the capacity budget; their absolute values fluctuate relatively more under non-Poisson inputs because of the small number of effective statistical samples, but the order of magnitude among priorities remains unchanged, and no target success rate is set for the second-highest and lowest priorities in this paper, so the conclusion of this section is not affected. In terms of throughput, the maximum difference among the four arrival processes over the six segments is 0.105 Mbps (Pareto α = 1.5 in segment R6), which is of the same order as the within-segment standard deviation of repeated simulations (0.022–0.068 Mbps).

The above results show that the engineering value of the proposed algorithm does not depend on the Poisson arrival assumption: under Pareto heavy-tailed and periodic arrivals, source-end probabilistic admission remains effective in guaranteeing the highest priority and in constraining the capacity, and the analytical bound derived under the Poisson assumption is still applicable.

#### 5.2.3. Algorithm Comparison Analysis: 16-Node Four-Channel

To verify the applicability of the proposed mechanism under a larger network scale and multi-channel conditions, this section further compares the performance of the four algorithms under the six-segment load scenario shown in Table 6.

**Transmission Success Rate (Metric A)**

**Figure 21:**
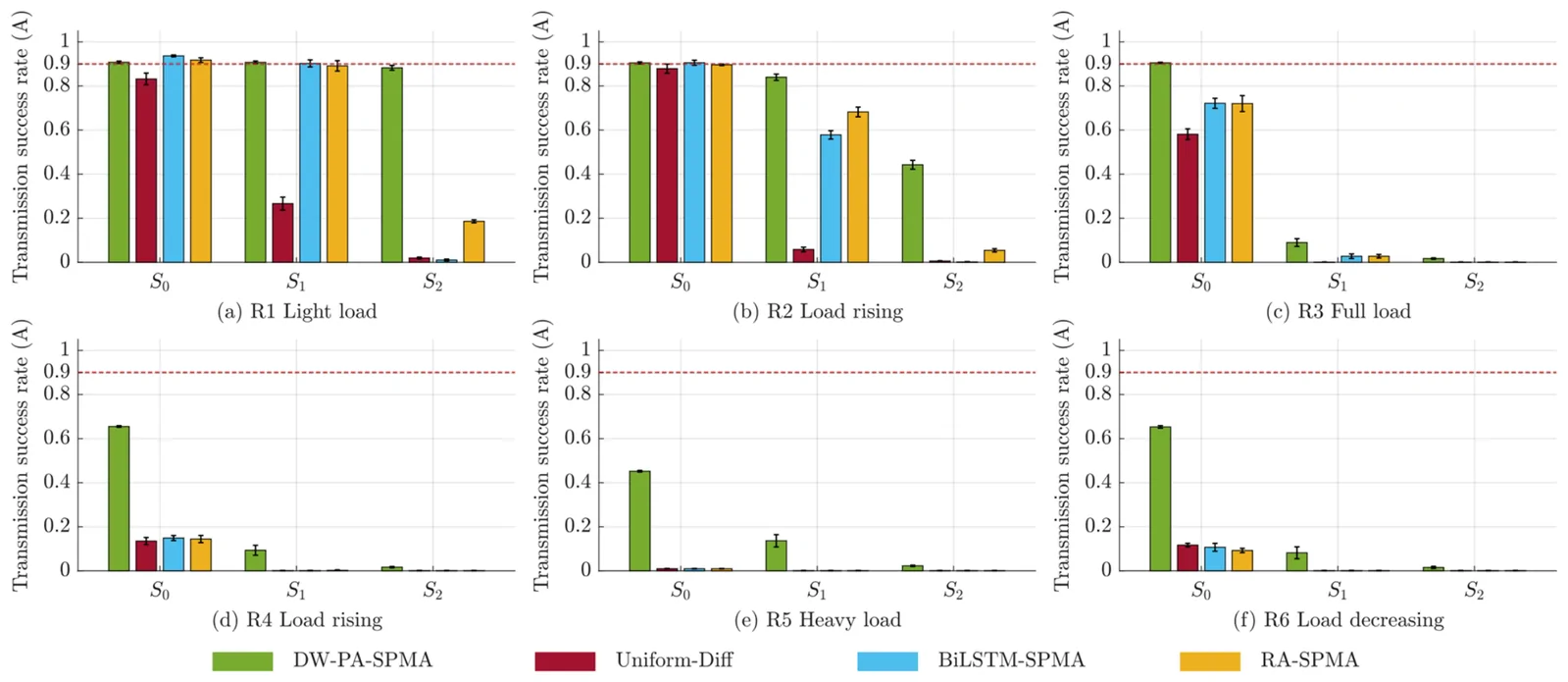
Comparison of transmission success rate (Metric A) across different load segments (16-node, four-channel).

Figure 21 gives the comparison of the Metric A transmission success rates of the four algorithms over the six load segments. For DW-PA-SPMA, the Metric A of the highest-priority service in the light-load to full-load segments (R1–R3) is 0.908, 0.905, and 0.905, respectively, comparable to the results in Section 5.2.2 (0.905–0.916), and still stably satisfies the 90% target; after entering the 2.0× heavy-load segment (R5), source-end probabilistic admission intercepts excess arrivals at the source and the Metric A drops to 0.452, consistent with the level under the single-channel condition. The service capability of the low-priority services shows the same pattern as in the single-channel case: in the full-load segment (R3) the Metric A of the second-highest and lowest priorities is about 0.09 and 0.02, respectively. As a comparison, the Metric A of the three comparison algorithms in the heavy-load segment (R5) all drops to about 0.01, that is, almost no highest-priority packet is successfully delivered to the application layer.

**Transmission Success Rate (Metric B)**

**Figure 22:**
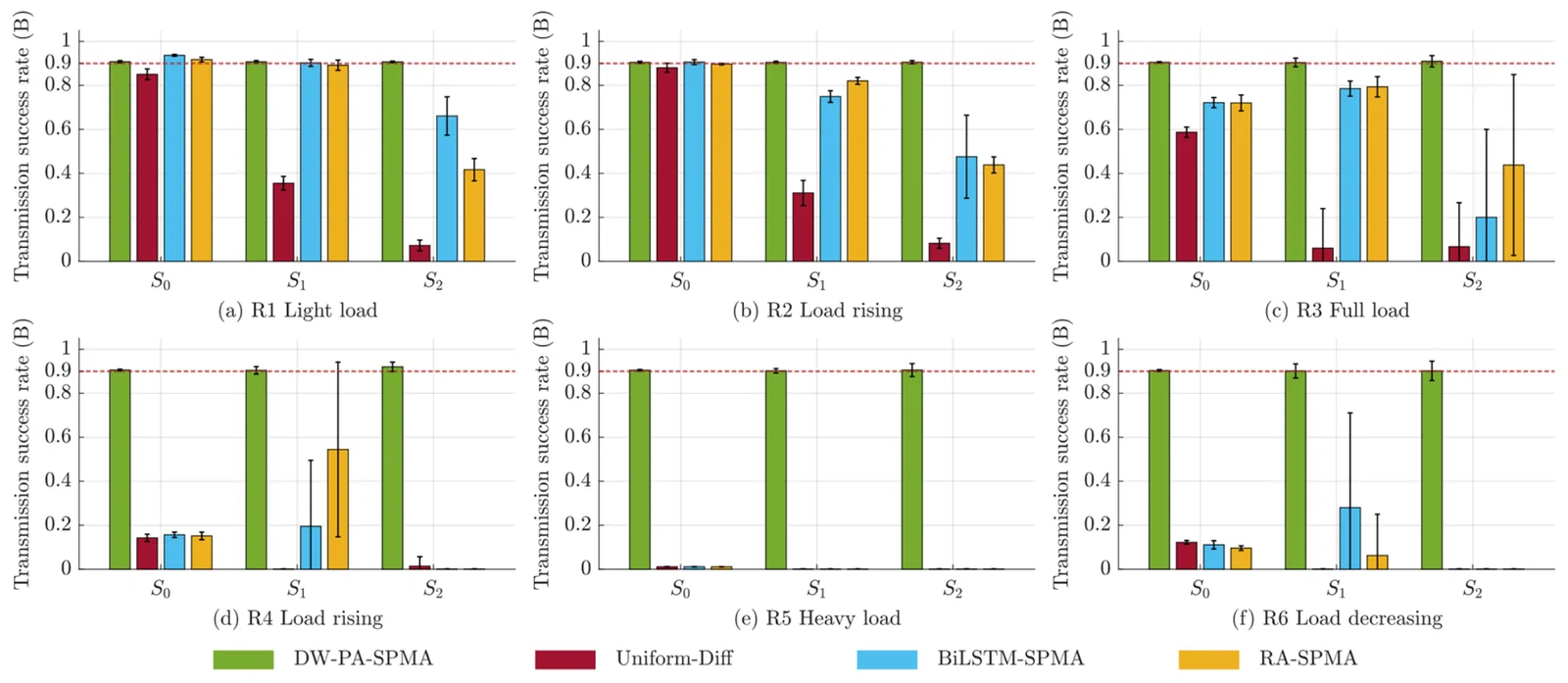
Comparison of transmission success rate (Metric B) across different load segments (16-node, four-channel).

Figure 22 gives the comparison of the Metric B transmission success rates in the same scenario. The Metric B of the highest-priority service of DW-PA-SPMA remains stable at 0.904–0.908 in all six load segments: that is, in the 2.0× heavy-load segment (R5), although the Metric A drops to 0.452, the transmission quality of the packets already entering the channel does not deteriorate (the Metric B in this segment is 0.905), indicating that source-end probabilistic admission constrains the traffic injected into the channel within the capacity budget and the intra-channel collision level does not deteriorate with the external arrival volume. As a comparison, Metric B of the three comparison algorithms in the light-load segment is 0.851–0.937, decreases markedly after entering the full-load segment, and drops to about 0.011 in the heavy-load segment (R5), indicating that their packets flood into the channel in large numbers and the collisions are extremely severe.

**End-to-End Delay**

**Figure 23:**
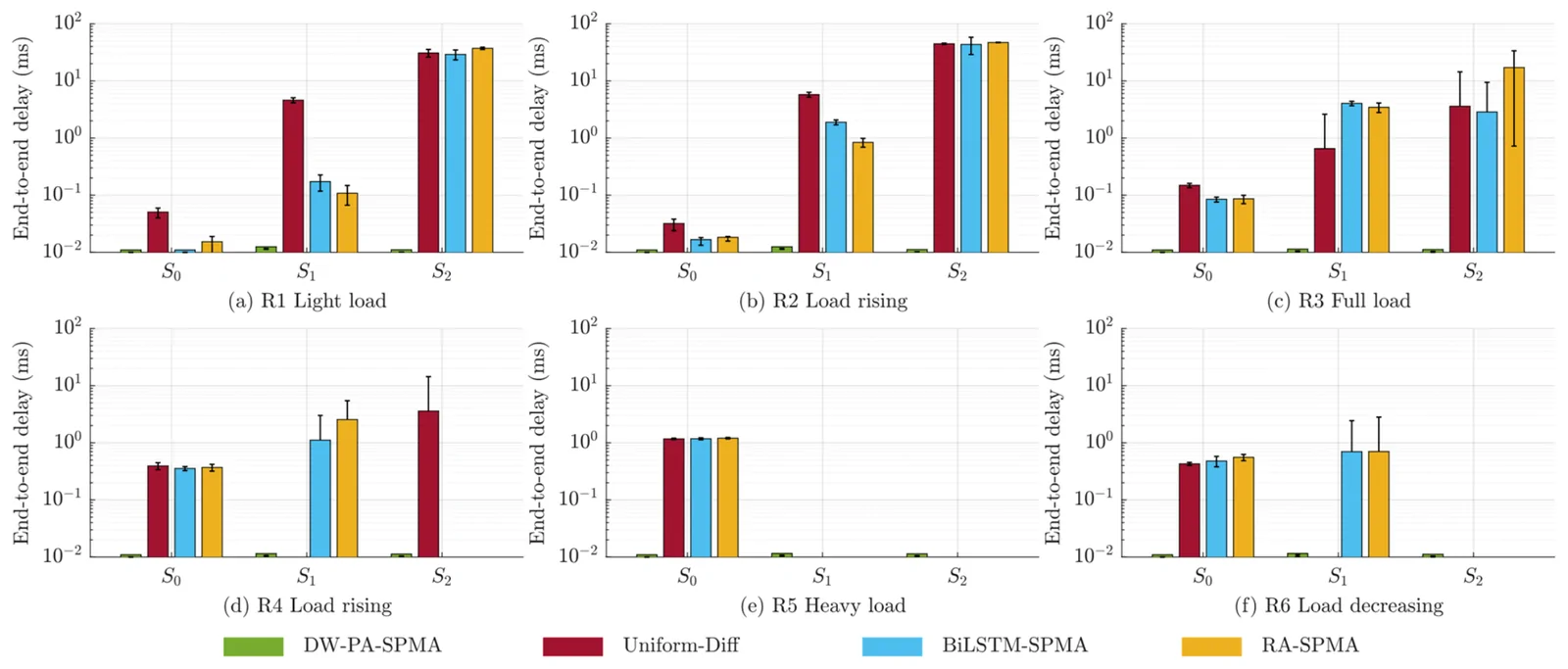
Comparison of end-to-end delay across different load segments (16-node, four-channel).

Figure 23 gives the comparison of the end-to-end delay (logarithmic scale). The end-to-end delay of DW-PA-SPMA in all load segments and for all priorities remains stable at 0.010–0.012 ms (about one time slot), consistent with the results in Section 5.2.2, verifying that the “admission means transmission” scheduling mechanism also holds under a larger scale and multi-channel conditions. As a comparison, the delay of the highest-priority service of the three comparison algorithms deteriorates rapidly as the load rises: 0.010–0.050 ms in the light-load segment (R1), 0.084–0.148 ms in the full-load segment (R3), and 1.171–1.203 ms in the heavy-load segment (R5), where the low-priority services have almost no successfully received packets.

**Throughput**

**Figure 24:**
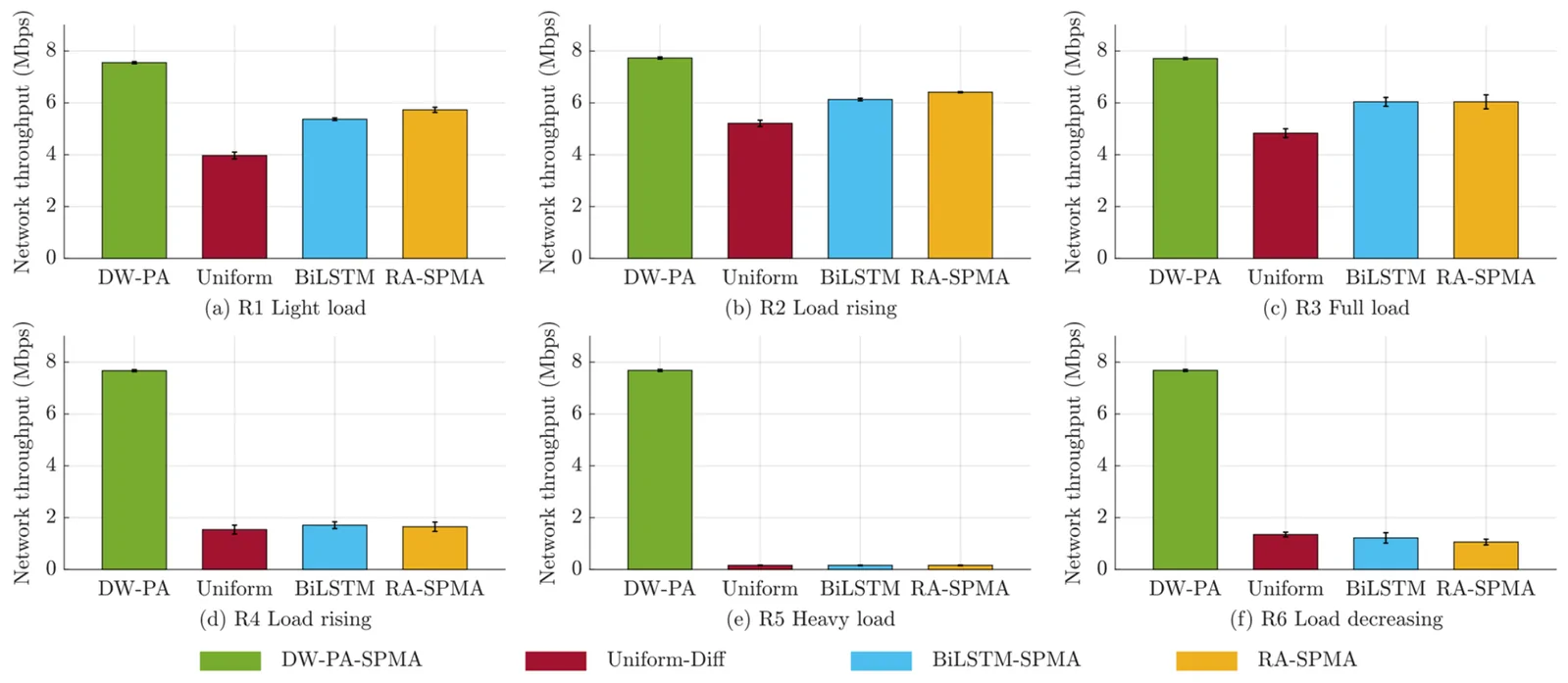
Comparison of throughput across different load segments (16-node, four-channel).

Figure 24 gives the comparison of the network throughput of the four algorithms. The throughput of DW-PA-SPMA in the light-load to full-load segments (R1–R3) is 7.55, 7.73, and 7.71 Mbps, after which it stabilizes at 7.67–7.68 Mbps and no longer climbs with the upper-layer traffic arrivals. The mechanism is consistent with Section 5.2.2: when the traffic exceeds the channel capacity limit, excess packets are dropped at the source and the total traffic entering the channel is constrained within the channel capacity budget, so the throughput reaches a saturation plateau. As a comparison, the throughput of the three comparison algorithms in the light-load to full-load segments is only 3.97–6.41 Mbps, and after entering the heavy-load segment (R5) they successively collapse to about 0.16 Mbps, only about 2% of the proposed algorithm, with channel resources heavily occupied by invalid packets.

The above results show two points. First, the performance advantage of the proposed mechanism does not depend on the specific configuration of 8 nodes and a single channel: when the network scale doubles and the number of channels increases fourfold, the baseline threshold, the thresholds at all levels, and the statistical windows can be converted synchronously with the network parameters according to Formula (2), and the mechanism can maintain the design target without any structural modification. Second, the performance bottleneck does not lie in the amount of physical resources but in the access control mechanism; adding channels merely provides more resources for the traditional mechanism to collide on, and cannot solve its structural problem of packets being first enqueued, then judged post hoc, and finally transmitted in bursts after accumulation.

## 6. Discussion

### 6.1. Algorithm Advantage Analysis

The performance gain of DW-PA-SPMA originates from the collaborative closed-loop design of statistical sensing, capacity budgeting, source-end admission, and scheduling transmission. Its core advantages can be summarized in three aspects.

First, the long–short dual-window architecture alleviates the inherent noise–lag dilemma of a single window. Single-window statistics must compromise between estimation noise and tracking lag: if the window is too small, instantaneous time-slot-level jitter directly interferes with decisions; if the window is too large, the tracking response to load changes is slow. This paper decouples the responsibilities: the short window completes aggregation denoising of instantaneous channel occupancy and does not directly participate in admission decisions; the long window is responsible for extracting steady-state load, exclusively for use by the capacity budget module. The division of labor between the two simultaneously suppresses statistical noise and takes into account load tracking capability, breaking through the one-dimensional compromise limitation of single-window parameters.

Second, changing traffic management from post-hoc to source-end probabilistic admission at the packet generation stage fundamentally eliminates the queue accumulation–aggregated collision positive feedback of traditional SPMA. The traditional threshold–backoff mechanism performs transmission interception only after packets are enqueued. Under overload, packets accumulate massively, and once the channel occupancy condition is satisfied, they are sent in a concentrated manner, destroying the Poisson transmission assumption and causing collision deterioration. This protocol analytically calculates the admission probability at each level based on the capacity budget. Excess packets are directly discarded at the source, without entering the queue or triggering backoff; by utilizing the Bernoulli sparsification invariance of the Poisson process, the packet flow after probabilistic screening still maintains Poisson statistical characteristics, so that the classical SPMA analytical model remains valid, and system performance is analytically tractable.

Third, “admission means transmission” scheduling eliminates queuing and backoff delay. Packets that have passed source-end admission no longer perform a second COS threshold check and are directly scheduled for transmission according to strict priority. The queue only stores admitted packets, so accumulation hardly occurs, and the end-to-end delay is stably constrained to a single time slot, with delay performance decoupled from the traffic load level.

The gain of the protocol comes from the integration of the above mechanisms rather than their simple superposition. If there were only probabilistic admission without accurate capacity estimation, the admission probability would lose its basis, which is exactly why traditional measurement-based methods must calibrate the baseline threshold offline. If there were only dual-window statistics without the admission mechanism, the improvement of statistical accuracy could not be converted into controllability under overload, because queues would still accumulate in the post-hoc interception mode. If there was only the adaptive long window without the capacity budget, the adjustment of the window length would lack a clear optimization objective. It is the integration of the three that makes the system form a closed loop of sensing, budgeting, admission, and scheduling: the statistics module provides the occupancy estimate, the budget module computes the admission probability of each priority accordingly, the admission module performs traffic shaping at the source, and the scheduling module realizes admission means transmission, while the transmission results are fed back to the statistics module. This closed-loop structure is the key difference between this paper and a simple combination of probabilistic access and thresholds, and is also the source of the performance gain.

The above four modules of sensing, capacity budgeting, source-end admission, and scheduling transmission do not work independently, but are linked at each time slot to form a complete closed-loop feedback. Load changes are sensed through channel occupancy statistics and automatically transmitted to the update of admission probability, without the need to design additional complex feedback parameter-tuning logic, ultimately achieving stable QoS guarantee for high-priority services under dynamic load scenarios.

### 6.2. Algorithm Applicability Boundaries and Improvement Directions

The proposed algorithm is not built from scratch. Source-end probabilistic admission and priority-based capacity allocation have been discussed in existing research; the methodological contribution of this paper lies in taking the analytical capacity budget framework as the core, integrating dual-window channel occupancy statistics with adaptive long-window control into a closed-loop admission mechanism that requires no prior load information, and providing complete performance verification and statistical characteristic analysis under time-varying load. In particular, the admission probability is neither a fixed constant nor an empirically set intensity parameter, but a derived quantity analytically computed from the traffic demand of each priority and the remaining available capacity after deducting the occupancy of higher priorities, forming a capacity budget chain in which each level yields to the next according to priority; this is the essential difference between the proposed method and existing probabilistic access mechanisms.

Compared with existing probabilistic access mechanisms, such as the fixed probability access of p-persistent CSMA and various traffic-shaping methods that probabilistically limit the access intensity, the differences in the proposed method lie in three essential aspects. First, the source of the admission probability is different. In existing methods, the access probability is usually a fixed constant or an empirically set intensity parameter, whereas in this paper the admission probability of each priority is analytically computed from the traffic demand of this priority and the remaining available capacity after deducting the occupancy of higher priorities by Formula (7), and is therefore a derived quantity that varies in real time with the traffic and channel state. Second, the layered structure is different. This paper computes the admission probabilities layer by layer according to priority: the admission probability of the highest priority is determined by its demand and the total capacity, the second-highest priority uses the remaining capacity after deducting the occupancy of the highest priority, and the lowest priority further deducts the occupancy of the second-highest priority, forming a capacity budget chain in which each level yields to the next, rather than imposing a single probability on all services. Third, the coupling with the statistical windows is different. The computation of the admission probability relies on the instantaneous and steady-state occupancy estimates provided by the dual-window statistics, while the long-window length itself is adaptively determined by the online-estimated load variation time scale, so that admission control and statistical sensing constitute a closed loop.

In terms of network form, the proposed algorithm is applicable to single-hop fully connected networks of any scale. The baseline threshold and the thresholds at all levels can be converted synchronously with the network scale and the number of channels according to Formula (2), and the algorithm itself requires no structural modification; in this paper, parameter calibration and sensitivity analysis are completed under the eight-node single-channel condition, and the same performance is verified under the 16-node four-channel condition, showing that its applicability does not depend on a particular network configuration. It should be emphasized, however, that the above applicability is built on the premise that a node can monitor the channel state of the whole network: in a single-hop fully connected network, all nodes share the same channel, the locally measured channel occupancy statistics are statistically equivalent to each other, and it is valid to estimate the whole-network channel occupancy level from local measurements, which is also the modeling premise of classical SPMA theoretical analysis. In a multi-hop network, however, different nodes have different neighborhood topologies, and local measurements cover only the range that the node can hear; treating them as the channel occupancy level of remote nodes may be either too large or too small, and the direction of the deviation depends on the distribution of transmitters within two hops. The existing local estimation correction method based on the ratio of 1-hop to 2-hop neighbor counts [20] is a topological statistic that cannot reflect the differences in real-time traffic load, and its estimation accuracy is poor. In future work, cross-layer information exchange will be introduced so that each node carries its locally measured channel occupancy statistic in its packets, enabling neighboring nodes to directly learn the channel occupancy level of their 1-hop neighborhood, thereby extending the proposed mechanism to multi-hop networks.

In terms of the traffic arrival process, the analytical bound and the admission probability computation in this paper are premised on the traffic arrival process of each priority being approximately Poisson, which determines the applicable condition of the capacity budget formula. The verification in Section 5.2.2 shows that when the traffic arrival process is changed from Poisson to a Pareto heavy-tailed distribution and to periodic arrivals, source-end probabilistic admission can still constrain the traffic injected into the channel within the capacity budget, and the transmission success rate (Metric A) of the highest priority in the full-load and heavy-load segments is essentially the same as under the Poisson input. This indicates that the computational structure of the capacity budget depends only on the mean arrivals of each priority and is independent of the specific form of the arrival distribution; this premise mainly affects the tightness of the analytical bound rather than the capacity constraint capability of the mechanism. Future work will further extend the arrival model to strongly bursty, strongly time-correlated, or mean-drifting traffic, and will quantify the residual influence of the higher-order statistical characteristics of the input stream on the post-admission output stream and its effect on the analytical bound; for the case where the traffic mean is in a slowly varying state that is difficult to observe, online estimation of the traffic volume will be introduced, which can share the same set of statistics as the long-window adaptive mechanism of this protocol.

## 7. Conclusions

This paper addresses the systematic problem in existing Statistical Priority-based Multiple Access (SPMA) protocols under dynamic load scenarios, where fixed thresholds and post-hoc backoff mechanisms cause severe deterioration of high-priority service transmission success rates, and proposes an improved SPMA protocol based on dual-window channel occupancy statistics and probabilistic admission control (DW-PA-SPMA).

The core contribution of this protocol lies in constructing a brand-new closed-loop “sensing–budgeting–execution–transmission” control architecture. First, through the long–short dual-window collaborative mechanism, it achieves accurate separation and independent statistics of instantaneous load jitter and long-term steady-state load for each priority service; second, based on this accurate sensing, it derives analytical calculation formulas for the available channel capacity and source-end admission probability of each priority, realizing a paradigm shift from “passive threshold interception” to “active capacity budgeting”; finally, through the proactive admission control module, it performs probabilistic shaping at the packet generation source, eliminating the positive-feedback congestion collapse caused by queue accumulation and random backoff in traditional protocols.

Simulation results verify the performance advantages of the DW-PA-SPMA algorithm. In a six-segment continuous time-varying service scenario containing light load, full load, and 2.0× heavy load, the algorithm maintains the transmission success rate of the highest-priority service above the 90% target from light load to full load, and in the 2.0× heavy-load segment the end-to-end completion rate remains at about 45% after source-end probabilistic admission shaping, while the three comparison algorithms are all below 1%. The parameter sensitivity analysis shows that when the short window is above the statistical denoising lower bound, the scaling-law coefficient of the long window is no greater than 0.5, and the calibration constant *K* and the dual-EWMA smoothing coefficients vary within a factor of two, the differences in the highest-priority transmission success rate are all at the level of the statistical fluctuation of repeated simulations, so the algorithm performance is insensitive to the above parameters; among them, the baseline threshold calibration deviation has a relatively direct effect and must be controlled within the calibration accuracy. Under the 16-node four-channel comparison test and the non-Poisson traffic of Pareto heavy-tailed and periodic arrivals, the algorithm maintains consistent design targets and constraint capability without any structural modification, demonstrating its potential to guarantee the service quality of core services in dynamic distributed networks.

Future research will focus on two aspects. First, the protocol will be extended to multi-hop network scenarios, where cross-layer information exchange and neighborhood cooperative sensing are introduced so that each node can learn the channel occupancy statistics of other nodes in its neighborhood, thereby overcoming the local estimation deviation caused by hidden terminals. Second, for traffic that deviates from the Poisson assumption, such as strongly bursty, strongly time-correlated, or mean-drifting traffic, the residual influence of its higher-order statistical characteristics on the post-admission output stream and the resulting deviation of the analytical bound will be quantified, and the applicable scope of the capacity budget mechanism will be extended in combination with online estimation of the traffic volume.

## Figures and Tables

**Table 1:** Summary of experimental results and causes for the four scenarios.

Scenario	Protocol and Traffic Configuration	Priority	Transmission Success Rate/Packet Loss Rate	Packet Loss Causes	Main Causes
1	ALOHA, single priority, full traffic load	*S*_0_	91.36%/ 8.64%	1. Collision failure ≈ 8.64% 2. Threshold interception: none	Natural collisions from Poisson random transmission, consistent with the preset target of 90%.
2	SPMA, single priority, full traffic load	*S*_0_	58.46%/ 41.54%	1. Collision failure ≈ 41.2% 2. Timeout/queue overflow ≈ 0.3%	COS fluctuates violently above and below the threshold, with 47.59% of time slots exceeding the threshold and being prohibited from transmission → packets are backed up due to backoff → concentrated transmission in low-occupancy time slots → collision probability surges. The main cause of packet loss shifts from “natural collision” to “aggregated collision triggered by threshold scheduling”.
3	SPMA, dual priority, each at full load, total overloaded	*S*_0_	9.35%/ 90.65%	1. Collision failure after sending ≈ 86.5% 2. Timeout/queue overflow ≈ 4.1%	*S*_1_ crowds into the sending gaps of *S*_0_, sending together with *S*_0_ packets in the same batch of low-occupancy time slots → collisions increase sharply. Fixed thresholds do not reserve capacity for *S*_0_, and the influx of *S*_1_ directly crushes *S*_0_.
		*S*_1_	0.21%/ 99.79%	1. Timeout/queue overflow ≈ 47.6% 2. Collision failure ≈ 52.2%	The *S*_1_ threshold is far below the COS fluctuation level, with only about 0.56% of time slots satisfying the transmission condition → packets cannot be sent for a long time, accumulate, and are discarded due to timeout; the few packets that obtain transmission opportunities also collide with other packets in aggregated bursts, almost all failing.
4	SPMA, dual priority, each at light load, total at full load	*S*_0_	81.13%/ 18.87%	1. Collision failure ≈ 18.9% 2. Timeout/queue overflow ≈ 0%	*S*_0_ packets can pass the threshold decision and be sent in most time slots; residual packet loss mainly comes from collisions caused by packets from different nodes being sent in the same batch of low-occupancy time slots.
		*S*_1_	13.03%/ 86.97%	1. Collision failure ≈ 68.3% 2. Timeout/queue overflow ≈ 18.6%	The *S*_1_ threshold is still below the normal level of COS, with only about 4.52% of time slots satisfying the transmission condition → packets cannot be sent for a long time, accumulate, and are discarded due to timeout; the few packets that obtain transmission opportunities are sent together with *S*_0_ packets in the same batch of low-occupancy time slots, resulting in severe collisions.

**Table 2:** Comparison of core mechanisms between traditional SPMA and DW-PA-SPMA.

Comparison Dimension	Traditional SPMA Protocol	Proposed DW-PA-SPMA Protocol
Load statistics window	Single fixed short window, unable to distinguish instantaneous fluctuation from long-term steady-state load.	Short window *W_s_* captures instantaneous COS as statistical input for the backend EWMA; long window *W_L_* smooths and extracts the long-term average load for each priority.
Traffic management location	Post-hoc: packets already enqueued, intercepted at transmission time slot through threshold decision.	Proactive: before enqueuing after packet generation, shaped at the source through probabilistic admission.
Management method	Threshold comparison + random backoff: packets not meeting the threshold remain in the queue, waiting for backoff retry.	Capacity-based probabilistic admission: admission probability *p_i_* computed according to available capacity *C_i_*, excess packets directly discarded, no backoff.
Load isolation capability	Global mixed statistics: only counts total COS of all services, cannot distinguish load of each priority.	Independent statistics per priority: accurately obtains ${\bar{\mathrm{COS}}}_{i}$ for each priority, enabling hierarchical capacity reservation and isolation.
Overload suppression capability	Weak: can only delay packet transmission, cannot suppress source generation, sustained overload leads to queue overflow and soaring collision rate.	Strong: clamps the long-term average load from the source within the baseline channel occupancy rate *COS_base_*, ensuring stable network operation under overload.
Scheduling stage operation	Repeated verification: COS must be compared with threshold before each transmission; backoff if not met.	No verification, direct transmission: admitted packets already guarantee capacity, no secondary verification needed during scheduling, achieving “admission means transmission”.
Delay characteristics	Load-sensitive: low delay under light load, queuing and backoff delay increase sharply under heavy load.	Load-insensitive: excess packets discarded at the source, queues barely accumulate, end-to-end delay constant at 1 time slot.

**Table 3:** Simulation parameter settings.

Parameter	Value	Parameter	Value
Number of nodes	8/16	Time slot length	10 μs
Number of priorities	3	Packet length	25 bytes
Number of channels	1/4	Priority queue length	100 packets
Simulation duration	200,000 time slots	Timeout (*S*_0_/*S*_1_/*S*_2_)	2/10/50 ms

**Table 4:** Five-segment load scenario parameters for the 8-node, single-channel network.

Load Segment	Time Slot Range	*S*_0_ Average Arrival Interval (Time Slots)	Load Level (Relative to Full Load)
R1 (light load)	0–40 k	152	0.5×
R2 (load increase)	40–80 k	152 → 76	0.5× → 1.0×
R3 (full load)	80–120 k	76	1.0×
R4 (load decrease)	120–160 k	76 → 152	1.0× → 0.5×
R5 (light load)	160–200 k	152	0.5×

**Table 5:** Six-segment load scenario parameters for the eight-node, single-channel network.

Load Segment	Time Slot Range	*S*_0_ Average Arrival Interval (Time Slots)	Load Level (Relative to Full Load)
R1 (light load)	0–30 k	152	0.5×
R2 (load increase)	30–60 k	152 → 76	0.5× → 1.0×
R3 (full load)	60–100 k	76	1.0×
R4 (load increase)	100–130 k	76 → 38	1.0× → 2.0×
R5 (heavy load)	130–170 k	38	2.0×
R6 (load decrease)	170–200 k	38 → 76	2.0× → 1.0×

**Table 6:** Six-segment load scenario parameters for the 16-node, four-channel network.

Load Segment	Time Slot Range	*S*_0_ Average Arrival Interval (Time Slots)	Load Level (Relative to Full Load)
R1 (light load)	0–30 k	76	0.5×
R2 (load increase)	30–60 k	76 → 38	0.5× → 1.0×
R3 (full load)	60–100 k	38	1.0×
R4 (load increase)	100–130 k	38 → 19	1.0× → 2.0×
R5 (heavy load)	130–170 k	19	2.0×
R6 (load decrease)	170–200 k	19 → 38	2.0× → 1.0×

**Table 7:** Transmission success rate (Metric A) of the highest priority for different values of the calibration constant *K*.

Value of *K*	R1	R2	R3	R4	R5
Baseline value (*K* = 50,000)	0.912 ± 0.007	0.907 ± 0.006	0.909 ± 0.004	0.915 ± 0.005	0.915 ± 0.007
Half the baseline value (*K* = 25,000)	0.913 ± 0.010	0.908 ± 0.006	0.912 ± 0.006	0.914 ± 0.005	0.912 ± 0.006

**Table 8:** Transmission success rate (Metric A) of the highest priority for different values of the dual-EWMA smoothing coefficients.

Value of the Smoothing Coefficient	R1	R2	R3	R4	R5
*τ_f_* = 100	0.910 ± 0.007	0.909 ± 0.008	0.913 ± 0.006	0.912 ± 0.007	0.910 ± 0.005
*τ_f_* = 200	0.915 ± 0.007	0.910 ± 0.007	0.909 ± 0.007	0.915 ± 0.007	0.911 ± 0.007
*τ_f_* = 400	0.912 ± 0.005	0.907 ± 0.006	0.907 ± 0.007	0.913 ± 0.006	0.914 ± 0.003
*τ_s_* = 2500	0.914 ± 0.007	0.910 ± 0.007	0.913 ± 0.006	0.916 ± 0.007	0.911 ± 0.009
*τ_s_* = 10,000	0.915 ± 0.005	0.910 ± 0.005	0.910 ± 0.005	0.912 ± 0.005	0.908 ± 0.007

## Data Availability

This study includes all of the data used in this work.
